# Regulation of Abusive Debt Collection Practices in the EU Member States: An Empirical Account

**DOI:** 10.1007/s10603-020-09476-8

**Published:** 2021-01-22

**Authors:** C.-G. Stănescu

**Affiliations:** Centre for Market and Economic Law, Faculty of Law, University of Copenhagen, Karen Blixens Plads 16, 2300 Copenhagen S, Denmark

**Keywords:** Debt-collection, Abusive practices, Member States, Code of Conduct, European Union, Consumer financial protection

## Abstract

The article seeks to establish, in a comprehensive manner, if and how abusive debt collection practices are regulated in the respondent EU Member States. Using empirical data gathered from consumer and supervisory agencies as well as debt collection associations in 26 EU Member States, it provides an insight into (a) the existence of a licencing regime for debt collectors; (b) the potential transboundary dimension of debt collection and its implications for the common market; (c) the types of abusive debt-collection practices encountered in the Member States; (d) the efficacy of self-regulation via Codes of Conduct; and (e) the potential traditional remedies available to consumer-debtors. The article concludes that the existence of different national models creates potential issues and discrepancies in the legal status and defences available to consumer-debtors across the EU, which ultimately affects the proper functioning of the single credit servicing market. The advocated solution is that of a harmonized sector-specific regulation of abusive debt collection practices at EU level.

The economic recession, the loss or decrease in income, and the reduction of welfare policies generated high levels of consumers’ over-indebtedness and affected their ability to repay their debts (Brown 2019; Deville 2015; Ferretti 2016). This in turn led to a surge in debt-collection cases. Ultimately, the Covid-19 pandemic intensified the problem of unmanageable personal debt. The pandemic caused loss of jobs in higher proportions than the Financial Crisis and further hampered economic recovery.^1^

Debt collection begins when a creditor determines that the consumer is behind with payments and must be contacted about repayment of the debt (Spanogle et al. 2013). When attempting to collect a debt, the creditor has the choice to resort or not to the help of the state’s apparatus (Smail 2016). Thus, debt collection may be divided into two categories: *judicial* and *non-judicial*, depending on whether state agents are involved or not.

The goal in debt collection is to recover the debt at the lowest possible cost, which is why judicial options may not be attractive due to the length, fees, and risks involved by the court procedure (Spanogle et al. 2013; Stănescu 2015; Warren et al. 2014). Therefore, most creditors will resort to non-judicial options trying to convince or pressure the consumer into repaying the debt.^2^ Non-judicial debt collection can become abusive if the creditor crosses the line of permissible conduct in order to extract payment from the debtor. Thus, for the purposes of this article, abusive non-judicial debt collection practices refers to all methods of enforcement employed by a creditor for debt recovery that do not involve the judiciary or other state agents (i.e., bailiffs, sheriffs, or police officers), and pose a threat to the consumer-debtor’s physical, psychological, or economic wellbeing.

Over-indebtedness affects mainly consumers and creditors, but they can also cause a ripple effect, reverberating through society and the local, regional or even global economy, with implications for legislators and policy makers. Consumer-debtors confront personal bankruptcy, social and financial exclusion, public shame, family or relationship hardship or psychological issues (Ferretti 2016; Spooner 2019; Warren et al. 2014), and are in a constant state of vulnerability (Jérusalmy et al. 2020). Meanwhile, creditors face disruptions in their activity and the risk of insolvency, which may cause them to engage in abusive practices in order to maximize collection and restore their economic situation.

A large-scale inability to pay, at both consumer and business level, can lead to wider economic consequences, affecting not only third party consumers or businesses, but also entire sectors or state finances. Ultimately, political and legal solutions will be needed to address the societal and economic challenges.

An example is the large number of non-performing loans (NPLs) in the EU caused by the 2008 Credit Crunch that led to a reduction in bank lending, which in turn affected negatively the EU’s economic growth (Macchiarelli et al. 2019). In response, the EU Commission presented in 2018 a package of measures designed to address the risks related to high levels of NPLs in Europe, including a Proposal for a Directive on Credit Servicers, Credit Purchasers and the Recovery of Collateral (NPLD Proposal 2008).^3^ The proposal aims to create a single market for credit servicing and the transfer of bank loans to third parties across the EU, and would have a major impact on a wide range of stakeholders: credit and non-credit institutions, debt buyers, debt collectors, and consumer-debtors (Huertas and Schelling 2020). Due to the large number of diverging legal interests, the implementation of this proposal has moved in slow pace (Zwitter-Tehovnik 2019), although things seem to have changed in the early months of 2020 (Huertas and Schelling 2020) likely due to the increasing over-indebtedness caused by Covid-19.

In late 2019, a number of amendments were put forward, the most important of which are contained in the European Parliament’s Committee on Economic and Monetary Affairs Draft Report (hereinafter CEMA 2019).^4^ The Draft Report makes a number of suggestions concerning activities related to debt collection. It starts by acknowledging the absence of common standards at European level to regulate debt collection practices (CEMA 2019, Amendments 229 and 230), and makes several proposals meant to achieve a sufficient level of borrower protection and address debt collection malpractice such as a ban on charging debtors the cost of debt-collection (CEMA 2019, Amendment 258) or a ban of aggressive, misleading or harassing practices (CEMA 2019, Amendment 336).

If the EU plans to deliver on its promise to ensure a high level of consumer protection,^5^ measures as those suggested in the amendments to the NPLD Proposal (CEMA 2019) must be implemented. In my view, the single market for NPLs is inextricably linked to debt recovery activities, with an emphasis here on non-judicial debt collection. NPLs make a growing market and attract investors due to their significant return opportunities. The more they recover, the more profit they make, which may encourage some debt collectors to engage in abusive practices in order to maximize recovery (also Jérusalmy et al. 2020, pp. 28, 33). This possibility needs to be counterbalanced by a high level of consumer protection against abusive debt-collection practices (Jérusalmy et al. 2020; Stănescu 2020).

Similar suggestions were made by the World Bank in connection to the deterioration of the consumers’ financial circumstances as a result of the Covid-19 pandemic. Among the immediate harms inflicted by the Covid 19 crisis on financial consumers due to unexpected loss of income, the financial institution listed the exposure of consumers to “*aggressive debt collection practices and late payment or default fees*” including seizure of social protection payments by debt collectors or financial institutions (Boeddu et al. 2020, emphasis added). Thus, in its recommended actions to regulators, the World Bank highlighted that “*enhanced monitoring of aggressive and unscrupulous debt collection activities is crucial during these times*” (Boeddu et al. 2020, emphasis added).

The initial NPLD Proposal indirectly recognized some of the risks, providing that credit servicers (debt collectors) should have a spotless criminal record in relation to serious criminal offences linked to crimes against property, crimes related to financial activities or crimes against the physical integrity, have a good reputation and meet professional standards to ensure that consumer will be treated fairly (NPLD Proposal, Recitals 24 and 53 corroborated with Art 5). Nevertheless, the proposal did not consider the need to harmonize fair debt collection practices across the EU, increasing the likelihood that in their attempt to maximize profit, debt buyers and debt collectors will engage in unfair behaviour against consumer-debtors. Moreover, it did not contemplate the likelihood that a cross-border market for debt portfolios with different standards of protection, varying from strict to no protection at all, might encourage regulatory or tax arbitrage and increase the risk of money laundering (Stănescu and Bogdan 2020).

So far, in the absence of a sector-specific legislation, the EU recommends the Unfair Commercial Practices Directive 2005 (UCPD 2005) to be applied to abusive debt collection practices. Based on an extensive interpretation of its scope,^6^ the UCPD 2005 enabled several Member States to sanction some misleading and aggressive debt collection practices. Notwithstanding all the above, the UCPD 2005 is rather unfit to effectively tackle abusive debt collection at the EU level. The UCPD 2005 was not purposefully intended to cover abusive debt collection practices in a comprehensive manner. Its effects in this area are mostly tangential and coincidental: It contains no rules concerning the authorization of debt collectors, no rules concerning the validation of the debt or the issue of added charges to the original debt (which are present in most sector-specific debt collection laws and were also referred to in the proposed amendments to NPLD Proposal), and, until the adoption of the Enforcement Directive 2019,7 it provided no individual remedy or private right to action to aggrieved consumer-debtors (Stănescu 2015, 2020). Moreover, its application to debt collection is impaired by a number of issues such as the conditions regarding the consequences of the unfair practices and the compliance with the average consumer standard (Stănescu 2020).

The suggested amendments to the NPLD Proposal have not yet been adopted or rejected at EU level. Nevertheless, some have expressed concerns regarding the (almost complete) absence of explanatory statements that could justify the above amendments (Huertas and Schelling 2020). This study aims to provide the necessary empirical background for their consideration.

## Previous Research

While scholarly works and empirical data, in connection with abusive debt collection practices are widely available in the USA, the same cannot be said about the EU. Academic works have covered extensively concepts such as money (Gleeson 2018), consumer credit (Brown 2019; Goode and McKendrick 2016; Goode 1989; Guest et al. 2009; Rosenthal and Haxton-Bernard 2018), security interests (Beale et al. 2012; Dahan and Simpson 2008; de Lacy 2010; Drobnig 2007; Rizoiu 2011; Tajti 2002, 2013, 2014), and the strong ties between them. However, debt collection has received very little attention (Deville 2015; Jérusalmy et al. 2020; Reifner et al. 2010; Tajti 2019; Stănescu 2015; Stănescu and Bogdan 2020).

So far, legal scholarship in English language has focused mostly on the major Western jurisdictions, such as France, Germany, or the United Kingdom (UK) (Deville 2015; Jérusalmy et al. 2020; Reifner et al. 2010; Stănescu 2015; Tajti 2019). Yet, these works are affected now by significant issues that diminish their relevance, such as reliance on erred, incomplete, or outdated^8^ information.

Recent studies (Jérusalmy et al. 2020) focus on the wider issue of human dignity of individual debtors in Europe, looking at various aspects such as household incomes and income protection, minimum income for a decent living in various European states, debt-collector malpractice, and debt advice. While this study is up to date and partially overlaps with the current project, it is significantly narrower in its coverage of abusive debt collection practices, focusing only on qualitative analysis of abusive debt collection practices experienced by some European states (both inside and outside EU). At the same time, the source of its information stems from local experts, while my study relies on answers received from consumer or supervisory agencies, and debt collection associations.

In the light of the above, it is necessary to conduct a thorough empirical investigation of all EU jurisdictions, in a holistic manner. This would provide both legislators and policy makers, as well as other stakeholders (consumer associations, the debt recovery industry, and legal researchers) with a clearer image of the current state of affairs in order to evaluate better the need for harmonization of regulation concerning abusive debt collection practices at the EU level, as proposed by the amendments to the NPLD Proposal (CEMA 2019).

### Article’s Aim and Hypothesis

This article seeks to establish, in a comprehensive manner, if and how abusive debt collection practices are regulated in the respondent EU Member States (EU MS). Subsequently, it provides an insight into (a) the existence of a licencing regime for debt collectors; (b) the potential transboundary dimension of debt collection and its implications for the single credit servicing market; (c) the types of abusive debt-collection practices encountered in the EU MS; (d) the efficacy of self-regulation via Codes of Conduct; and (e) potential remedies available to consumer-debtors. Finally, it aims to answer whether the current national legal frameworks suffice to tackle abusive debt collection, or a pan-EU solution is needed.

My hypothesis is that although non-judicial debt collection is widely used and known in all MS, it is a largely unregulated legal area, with a cross-border dimension. Furthermore, I venture that its idiosyncratic features make for a heterogeneous and fragmented legal framework, which might be an obstacle in tackling cross-border abusive debt collection. Ultimately, assuming that the hypothesis is confirmed, it should be possible to infer whether the EU needs a harmonized legal framework, or national solutions suffice.

### Participants

The article contributes to the existing body of literature on the topic with empirical evidence, gathered from twenty-six out of twenty-seven EU MS via two surveys. Initially, the project envisioned only communication with consumer agencies (state bodies or supervisory agencies). However, to ensure a balanced representation of interests, I decided to contact the debt collection associations active in the MS as well. Thus, data collection had two prongs: consumer or supervisory agencies and debt collection associations. The respondents were selected for having competence in the field as well as access to the information sought by the surveys. Moreover, since most respondents are subjected to rules concerning free access to information of public interest, the data obtained is deemed both valid and reliable.

One survey that was circulated with more than 167 consumer associations or organizations, supervisory agencies, and financial ombudsmen.^9^ The survey generated twenty-eight fully completed and sixteen partially completed responses, thus ensuring a wide and comprehensive coverage of the EU jurisdictions. No answers were received from Malta.^10^ Another survey was circulated with 69 debt collection associations in the EU.^11^ Thirteen of them provided fully completed responses, while two only made partial submissions. All answers received to the survey were uploaded to an electronic archive and will be made publicly available at the end of the project.

The respondents were requested to indicate and provide their up-to-date legislation, regulation or, respectively, their Codes of Conduct concerning abusive debt collection practices. Legislation available only in the national language of the MS was translated by authorized translators to facilitate understanding. It was used in this study mainly to clarify contradictory answers received via the survey and, partially, to address the lack of answers from some of the respondents where the legal provisions left no room for doubt or interpretations (such as licencing requirements, sanctions for abusive behaviour, or levels of fines). Even so, the use of legislation for this purpose was kept at a minimum, in order not taint the results of the study. An in-depth comparative doctrinal assessment of the existing national legislation concerning abusive debt collection in the EU MS will make the subject of a separate paper.

The received data has been verified against or corroborated with other data available, mostly taken from EU reports and assessments conducted at the request of the EU Commission, such as the Luxembourg Report on European Procedural Law (Hess and Law 2019), EU Justice Scoreboard (European Commission 2018),^12^ the Fitness Check of consumer and marketing law, and of the evaluation of the Consumer Rights Directive (European Commission 2017).^13^ It also resorted to national reports and studies, such as the Netherlands Authority for Consumers and Markets’ Study into the Commercial Practices of Debt Collection Agencies (NACM 2015). Ultimately, the study referred to previous academic work and consumer law and policy empirical reports (Jérusalmy et al. 2020; Nessel 2019).

### Ethical Concerns and Data Protection

The data sought by the survey was quantitative and fell under the coverage of public-interest information. No private or sensitive data was requested or generated by the survey and the received answers. Thus, the study raised no ethical or personal data protection related concerns.

### Survey Design

At the time when the project was designed, the EU had twenty-seven MS, and twenty-four official languages.^14^ I have used two standardized surveys, in English language, circulated via an email-service (SurveyXact). According to the project proposal, the surveys were expected to gather data from at least ten of the twenty-seven EU MS to advance knowledge in the field and allow a meaningful compilation and comparison of data. In the end, the surveys circulated with consumer agencies and supervisory bodies gathered data from twenty-six MS, while the one circulated with debt collection associations gathered data from ten MS. Thus, from the perspective of the expected representativeness, the project achieved its target having a success response rate of over 96% of all EU MS.

The survey circulated with supervisory bodies and consumer agencies had six themes, five corresponding to the building blocks for an efficient regulation of fair debt collection practices advanced by Stănescu on the basis of the Fair Debt Collection Practices Act in the US and the Consumer Credit Sourcebook in the UK (Stănescu 2015), plus an open-end question. Using these themes was justified by a number of reasons. First, the themes are deemed minimum core elements of any efficient regime dealing with non-judicial debt collection. Second, these themes can be identified in most sector-specific legislations tackling abusive debt collection practices. Third, the themes work beyond idiosyncratic differences and systemic approaches between various jurisdictions. Fourth, the themes allow for objective assessment of legislation having impact on non-judicial debt collection, independent of its wording or status. Finally, to date, there is no other theoretical framework available for the analysis of abusive debt-collection practices regimes.

The five themes were (a) the existence, scope, and coverage of sector-specific legislation as well as the usage of UCPD 2005 in the respondent MS (Questions 1–4); (b) the general status of licencing and supervision of debt collection entities, independent of whether their activity was subjected to a code of conduct or not (Questions 5–6); (c) the number and type of complaints received by each entity and the type of abusive debt collection practices reported (Questions 7–11); (d) the potential obligation of the debt-collector to validate the claim and the right of debt collectors to levy extra-charges for the collection activities (Questions 12–14)^15^; (e) the remedial and enforcement options available to consumer-debtors that were subjected to abusive debt collection practices^16^ (Questions 15–26). The open-end question (Question 27) concluded the survey, allowing respondents to make comments or recommendations on improving the regulation of abusive debt collection practices. A secondary survey was designed along the same lines for debt collection associations. The main difference between the two surveys was that the secondary survey sought also to collect data about the existence of Codes of Conduct at national level, their provisions, and their enforcement.

The surveys’ contents were discussed with the projects’ supervisor and peers at the University of Copenhagen. It was subsequently submitted for feedback to the Danish Competition and Consumer Authority (Konkurrence- og Forbrugerstyrelsen), the Danish regulatory and supervisory body, and to the Danish Consumer Council (Forbrugerrådet Tænk), a Danish independent consumer NGO due to their expertise in consumer law and wider experience with data collection and analysis.

### Data and Data Collection

The collected data is mostly quantitative. The goal was to provide an overview of the current legal framework concerning abusive debt collection practices in the EU MS, identify the commonalities and idiosyncratic features, and use the results to answer the research questions.

It should be mentioned that while consumer agencies and supervisory bodies represent *public, general interests*, and are obliged to disclose information of relevance to the public, which can be easily verified, the debt collection associations represent *private, industry interests,* and are not subjected to similar rules; their answers are voluntary in nature. Put differently, the latter’s answers may reflect bias and may have a larger degree of inaccuracy, for which reason, in the final assessment, they were used mainly for corroborative purposes.

Since several respondent MS have provided multiple answers, via different bodies, it can be inferred that the information received from them is more reliable. However, in the data analysis phase, I identified several instances where respondents from the same MS provided contradictory answers. I will explain how these were handled in the relevant sections.

### Methodology

The main purpose of the article is to provide a comprehensive state of the art concerning the regulation of abusive debt collection practices in the EU. To this end, the article resorts primarily to *empirical research,* as all its conclusions draw from verifiable evidence obtained via methods such as surveys and open-end questions. The empirical analysis falls into the category of a holistic, descriptive study that aims to provide data on the status quo in the surveyed MS, to present and categorize the current status and emphasize the features of non-judicial debt-collection regulation in the EU. The data collected via the surveys addressed to a large number of respondents are, thus, quantitative and help determine *who, what, where and how* regarding the regulation of abusive debt collection practices in the respondent EU MS, but do not allow to establish any causality (the *why*), which remains to be addressed in future research.

Elements of *comparative* (the analysis draws on information coming from multiple EU jurisdictions)*, and theoretical* (the analysis does not end with a mere determination of what the law is, but seeks to determine whether legal reform or intervention is necessary and desirable) research can also be identified, although their role remains limited*.*

## Results

The results are grouped, presented, and discussed on categories of questions.

### General Status of Sector-Specific Legislation and Applicability of UCPD to Abusive Debt Collection Practices

At the first question, on whether the respondent’s MS has sector-specific legislation addressing abusive non-judicial debt collection practices, I received answers, from twenty-six out of twenty-seven MS.

According to the replies, nine MS have sector-specific legislation: Belgium, Denmark, Finland, Germany, Greece, Latvia, the Netherlands, Romania, and Sweden. Seventeen MS indicated that they do not have such legislation in place.

Four MS require clarifications. Austria has legislation dealing solely with occupational licencing of debt-collectors. Since the law does not cover abusive debt-collection practices and the respondent consumer organizations indicated there is no such legislation in place, Austria was not counted among countries with tailored made legislation. France does have rules regarding formal requirements for written communication with consumer-debtors in a chapter of their Code of Civil Procedure (Stănescu 2015), which was deemed by the debt-collector associations “sector-specific legislation.” However, the supervisory body answered in the negative and given the general nature of the Civil Code of Procedure (*lex generalis*) the latter’s answer was given preference. In Hungary, the Central Bank issued a Guideline on debt recovery, which is not-binding and falls into the category of soft-law (Tajti 2019). Given the non-binding character of the Guideline and the negative answer of the supervisory body, the survey did not include Hungary among the states with sector-specific legislation. Finally, Malta provided no data, but there were strong indications it does not have sector-specific legislation either.

**Fig. 1:**
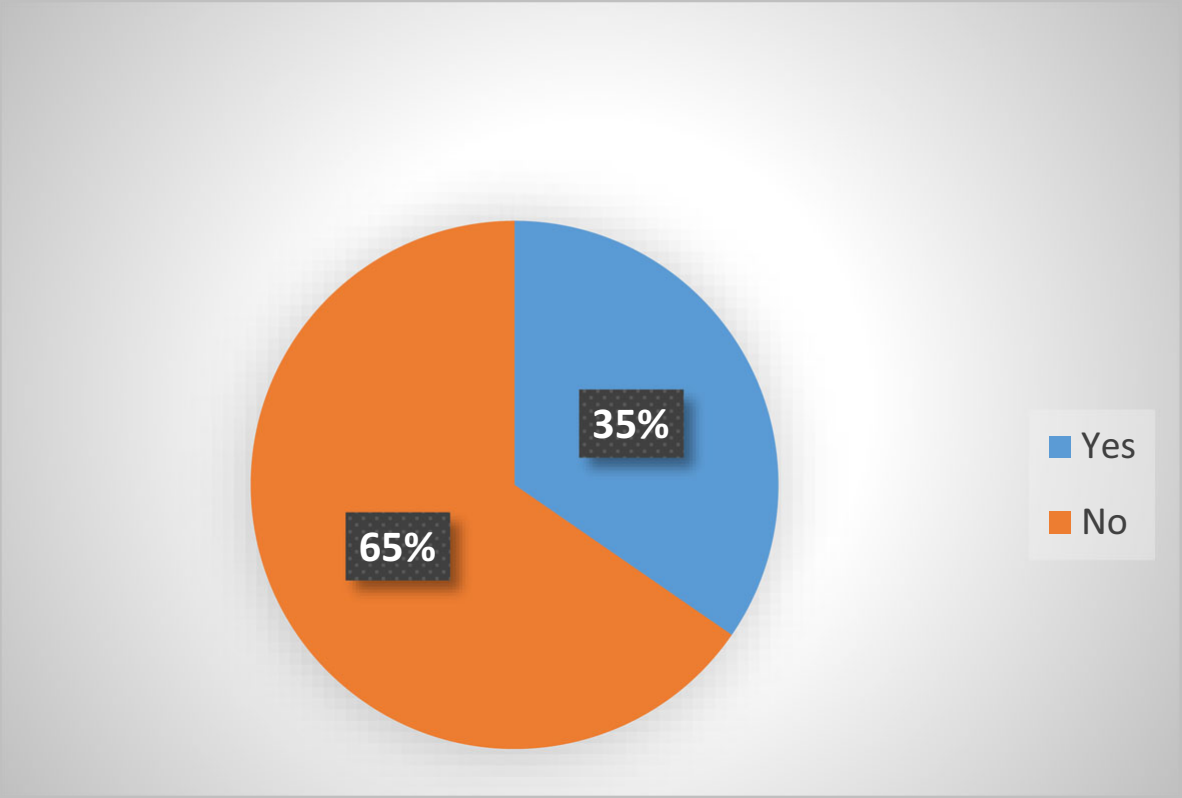
Number of MS with sector-specific debt collection legislation—26 MS

Based on the number of MS, it appears that around two thirds of EU MS does not provide specific protection against abusive non-judicial debt collection practices. This confirms prior assumptions (Jérusalmy et al. 2020, p. 4; Stănescu 2015) that debt collection is largely unregulated (Fig. 1).

**Fig. 2:**
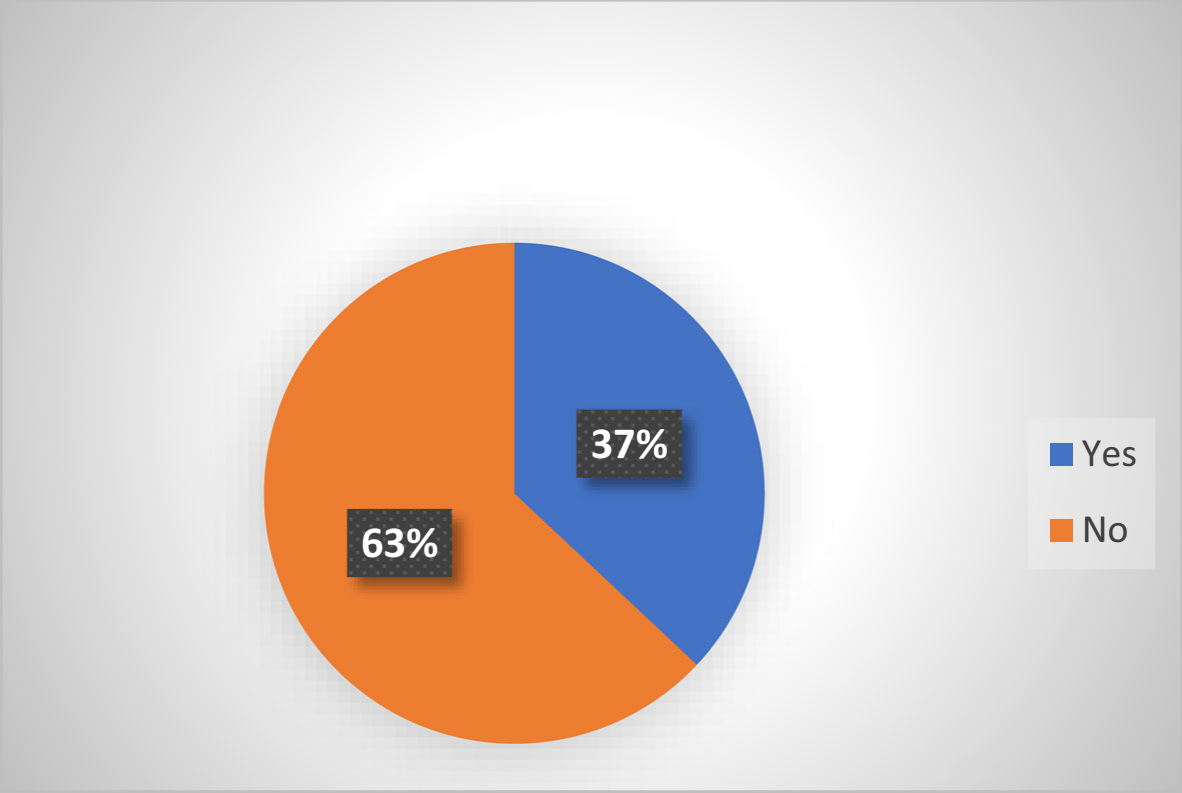
Sector-specific legislation in 27 MS (excluding UK) by population

A more precise evaluation of the number of consumers potentially exposed to abusive practices could be determined by taking into consideration the population number of each MS. The total number of consumers in the EU^17^ is 446.82 million. These figures refer to the total number of population, including minors. Although minors might not always qualify as consumers, they are still considered here as part of the debtor’s “close circle,” which exposes them to abusive debt collection practices, for which reason they were included in the count. Out of these, 281.47 million consumers live in MS that do not have sector-specific legislation. In other words, if one considers the current EU population, almost two thirds (63%) does not enjoy tailored protection against abusive non-judicial debt collection practices at MS level (Fig. 2).

As one may easily notice from the attached graphs, the percentages are consistent, because the percentage of MS that do not have a sector-specific legislation (65%) and the number of consumers living in MS that do not have a sector-specific legislation (63%) are very close. This should put in perspective for both national and the EU legislators the existing disparities in protection against abusive debt-collection at MS level via tailored-made legislation. The wide absence also underlines the importance of potential alternatives, such as EU legislation (UCPD 2005) or traditional remedies (general civil, commercial or consumer protection law).

**Fig. 3:**
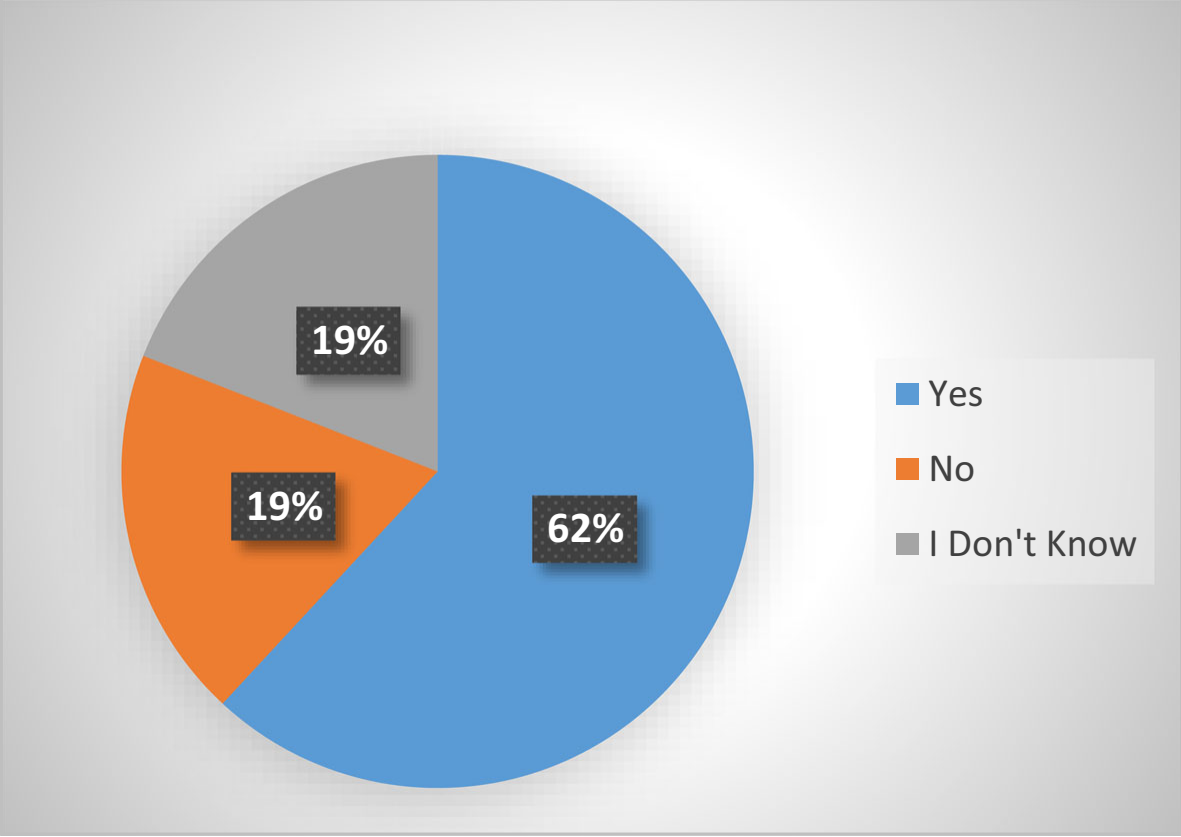
Use of UCPD in 21 respondent MS

According to the EU Commission, abusive debt collection practices are covered by the UCPD 2005 (European Commission 2016). This would mean that it may be possible to provide protection to aggrieved consumer debtors via the provisions of UCPD 2005 (and its national transpositions). Thus, the survey sought to determine how many of the MS resort to the UCPD (Fig. 3).

Out of twenty-one respondents, thirteen declared that the UCPD 2005 is used in their MS to tackle abusive debt collection practices (Belgium, Czech Republic, Denmark, Estonia, Finland, Germany, Italy, Latvia, Netherlands,^18^ Poland, Slovakia, Slovenia, and Spain). Four answered negatively (Bulgaria, Ireland, Luxembourg, and Sweden), while other four answered they do not know (Austria, Croatia, Lithuania, and Portugal).^19^ No data, due to lack of answers, was obtained from Cyprus, France, Greece, Hungary, Malta, and Romania.

Three aspects are worthy of attention. Firstly, many of the respondent MS that resort(ed) to UCPD 2005 also have sector-specific legislation in place (Belgium, Denmark, Finland, Germany, Latvia, and Netherlands). At the same time, only seven out sixteen MS lacking sector-specific legislation appear to use the UCPD 2005 as an alternative (Czech Republic, Estonia, Italy, Poland, Slovakia, Slovenia, and Spain). The results are consistent with those provided by the EU Commission’s 2016 Guidance on the UCPD 2005, which nominated Italy, Poland, and Slovakia to illustrate the UCPD 2005’s fitness in tackling abusive debt collection^20^ and with the Fitness Check, (European Commission 2017, pp. 47, 252–253).

Secondly, the number of respondents that indicated they do not know whether the UCPD 2005 is used, or ignored the question, should raise concern about the awareness of local agencies and the knowledge of their personnel of consumer-debtor remedies and protections available under EU law. A factor explaining this phenomenon may be that some of them are new MS, who may lack resources or qualified personnel or may have little experience in the field (Croatia, Estonia, and Lithuania). This explanation is not entirely satisfactory in case of Austria and Portugal, which are longstanding members of the EU. In their case, the low awareness might stem from the fact that non-judicial debt collection is a niche area of financial services.

Thirdly, four MS have answered in the negative and while their answers may be affected by personal knowledge, it may also be an indication of the fact that the UCPD 2005 is not always deemed fit to deal adequately with abusive non-judicial debt collection practices. Other explanations are possible, such as the presence of sector-specific legislation (in Sweden) or the use of other type of legislation or general law remedies (in Ireland). That abusive debt collection practices might not be considered a problem in these MS was also considered as a potential explanation, but it was dismissed since all four indicated the existence of complaints in this regard.

### General Status on Licencing and Supervision of Debt Collection Entities

As a first or even only line of defence against unscrupulous debt-collectors,^21^ licencing and supervision of their activity are matters of utmost importance for aggrieved consumer-debtors, consumer agencies or NGOs and enforcement bodies. Thus, it was important to determine how many MS require debt collectors to obtain some sort of occupational licencing, before being allowed to operate, even in the absence of sector-specific legislation.

**Fig. 4:**
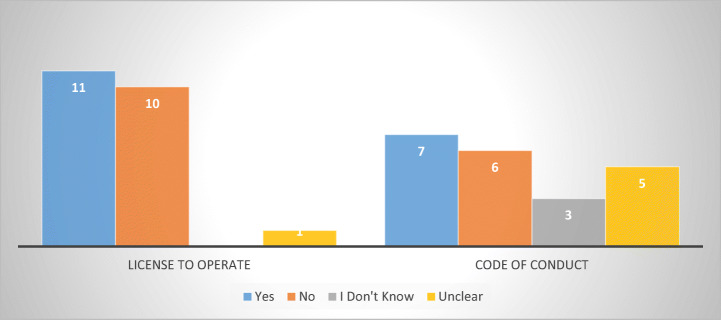
Licence to operate and Codes of Conduct—22 MS

The survey revealed that out of twenty-two respondents, eleven licenced the activity of debt-collectors (Austria, Belgium, Denmark, Finland, France, Germany, Greece, Italy, Latvia, Romania, and Sweden,).^22^ At first glance, most of the MS that require debt-collectors to have a licence to operate are those with sector-specific legislation in place, with the notable absence of Netherlands and the additions of Austria, France, and Italy.^23^ What is clear, nonetheless, is that in an almost equal number of MS (Bulgaria, Croatia, Czech Republic, Estonia, Ireland, Luxembourg, Lithuania, the Netherlands, Poland, and Slovenia), debt-collectors operate without any kind of tailored-made state supervision (other than that set by traditional general law remedies) (Fig. 4).

The benefit of licencing is to ensure that national authorities can supervise their activity and compliance with national legislation and/or Codes of Conduct, as well as to enable consumer-debtors aggrieved by unscrupulous businesses to file an administrative complaint against them. However, such benefit is hindered, especially in a cross-border context, if the empowered national authorities vary or have either overlapping or divided roles. Thus, the survey sought to determine which authorities have competence in issuing licences and monitoring the debt-collector’s activity. The answers summarized in the table below indicate a large variety of institutions and different competent bodies in MS, which makes the legal framework difficult to navigate for non-local consumers, a matter highlighted by other empirical studies (Table 1) (Hodges 2019, p. 197–200). It also highlights the idiosyncratic features of each national system, which points to the need to either harmonize the institutional framework, or the standard of protection at EU level (for concurrent conclusion, Hess and Law 2019, p. 5).

**Table 1:** Large variety of institutions and different competent bodies in MS

Member State	National Authority for Licencing and Supervision of Debt Collection Agencies According to Consumer Agencies and Supervisory Bodies	National Authority for Licencing and Supervision of Debt Collection Agencies According to Debt Collectors’ Associations
Austria	None	Gewerbebehörde (Trade Authority) (for licencing); Gewerbebehörde and Austrian Economic Chambers (for supervision)
Belgium	Ministry of Economic Affairs (for licencing); Economical Inspection (for supervision)	Ministry of Economic Affairs (for licencing and supervision)
Bulgaria	None	None
Croatia	None	*No data available*
Czech Republic	None	None
Cyprus	*No data available*	*No data available*
Denmark	National Chief of Police (licencing); National Chief of Police and the Consumer Ombudsman (supervision)	*No data available*
Estonia	None (for licencing); The Consumer Disputes Committee (the Estonian ADR), the Estonian European Consumer Centre and the Consumer Protection and Technical Regulatory Authority (general supervision)	*No data available*
Finland	The Regional Administrative Agency of Southern Finland (for licencing); The Finnish Competition and Consumer Authority (Kilpailu- ja kuluttajavirasto); the Consumer Ombudsman (kuluttaja-asiamies); The Regional Administrative Agency of Southern Finland (Etelä-Suomen aluehallintovirasto); (possibly the Financial Regulatory Authority - Finanssivalvonta); the Consumer Disputes Board (for supervision)	*No data available*
France	*No data available*	Prefecture of Police (for licencing)
Germany	Präsident of District Courts (Amtsgerichts) (for licencing & supervision);	Courts, depending on the states
Greece	*No data available*	*No data available*
Hungary	*No data available*	*No data available*
Ireland	None (for licencing); The Police (Garda), The Central Bank of Ireland, The Competition and Consumer Protection Commission (for supervision)	*No data available*
Italy	Regional Police (for licencing); Antitrust and Privacy Authority (for supervision)	Ministry of Interior (for licencing)
Latvia	The Consumer Rights Protection center (for licencing and supervision)	*No data available*
Lithuania	None	*No data available*
Luxembourg	None	*No data available*
Malta	*No data available*	*No data available*
Netherlands	None (for licencing); The Netherlands Authority for Consumers and Markets, Authority for Financial Markets (for supervision)	*No data available*
Poland	None	None
Portugal	*No data available*	
Romania	The National Authority for Consumer Protection (for licencing and supervision)	The National Authority for Consumer Protection (for licencing and supervision)
Slovakia	The National Bank of Slovakia (for licencing); Slovak Trade Inspection (for supervision)	*No data available*
Slovenia	None (for licencing); Market Inspectorate of the Republic of Slovenia, Police (for supervision)	*No data available*
Spain	*No data available*	*No data available*
Sweden	Swedish Data Protection Authority (for licencing and supervision)	Datainspektionen (The Swedish Data Protection Authority) (for licencing); Swedish Data Protection Authority, the Swedish Consumer Agency (for supervision)

Another aspect to determine was whether and how many debt collectors must abide to a Code of Conduct, at company, association, or industry level. According to the collected data, seven respondent MS claimed that debt collection must abide to a Code of Conduct to obtain a licence. Out of these, two stated that the Code of Conduct was required at industry level (Finland and Italy), two at company level (France and Latvia) while three (Belgium, Denmark, and Sweden) reported that the regulatory body imposed the Code of Conduct. Five MS provided contradictory answers that could not be verified (Austria, Bulgaria, Netherlands, Poland, and Slovakia), while three answered they are not aware of such requirement (*supra* Fig. 4).

While the data is non-exhaustive and may be prone to corrections, one must note the limited role played by Codes of Conduct in connection to abusive debt collection practices. Given that a significant number of respondents either indicated their absence or were unaware of their existence, suggests that Codes of Conduct cannot replace sector-specific legislation in the assessment and complaint handling of potential complaints, which confirms similar hypotheses (Jérusalmy et al. 2020, p. 28).

### Number and Types of Complaints Concerning Abusive Debt Collection Practices

**Fig. 5:**
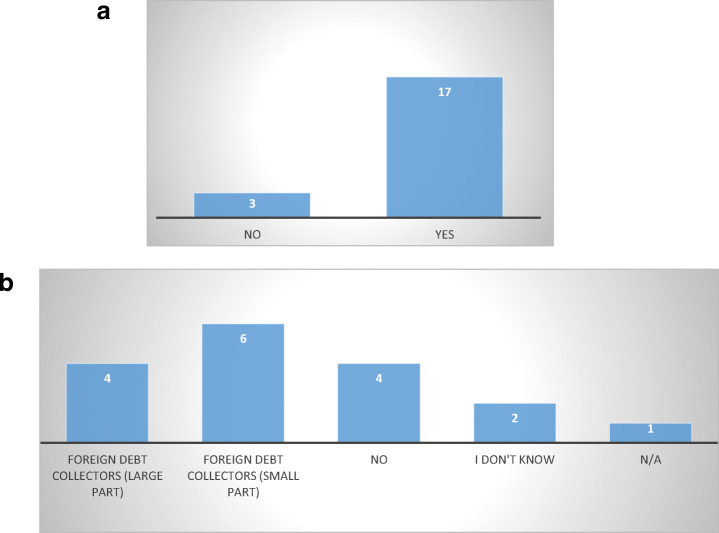
**a** MS that have received complaints concerning abusive debt collection practices in the past 5 years 20 MS. **b** Complaints concerning foreign debt collectors—17 MS

To evaluate the dimension of the debt collection issue in the EU (at both national and cross-border levels), respondents were asked if and how many complaints they received in the past five years in connection to abusive debt collection practices, indicating also if and how many of these involved foreign debt collection entities. Furthermore, respondents were asked to mention what types of abusive practices were reported, to get an estimate of which practices are more common and, thus, more problematic (Fig. 5).

The survey asked for estimates, as no figure would reflect the full reality of abusive debt collection practices in a particular MS. Firstly, the number of complaints received by each respondent institution did not necessarily mean those were all the complaints made in the respondent MS. Secondly, the number of received complaints did not mean an equal number of abuses, an aspect that depends on the merits of each case. Thirdly, many of the abusive practices go unreported. Thus, results presented here reflect only what consumers perceived as an abusive practice, to the extent that they would file a complaint about it.

The answers confirm, however, that abusive debt-collection practices are a widespread issue across MS (Jérusalmy et al. 2020, pp. 33–34). Seventeen out of twenty respondent MS indicated they have received complaints thereof in the past five years, compared to only three that indicated the opposite (Austria, Ireland, and Lithuania), while six respondents provided no answer to the question. However, the negative response should not be interpreted in the sense that no abusive practices occurred in these MS, but that the respondent body did not receive any.

About complaints against foreign debt collectors, four respondents indicated that they were the subject of a large number of complaints (Belgium, Czech Republic, Luxembourg, and the Netherlands), while other six indicated a small number (Bulgaria, Estonia, Finland, Germany, Latvia, and Sweden).

Notwithstanding the potential imprecision stemming from the numbers collected, two matters are undeniable. Firstly, most MS experience consumer-complaints regarding abusive debt-collection practices, to a greater or smaller extent. Secondly, many of these complaints are directed at foreign debt collectors, which highlights the cross-border dimension of the issue, and, given the regulatory void mentioned above, the potential for a regulatory arbitrage, by registering in a MS with lax or no supervision and then taking advantage of the EU freedoms to conduct business across the union.

**Fig. 6:**
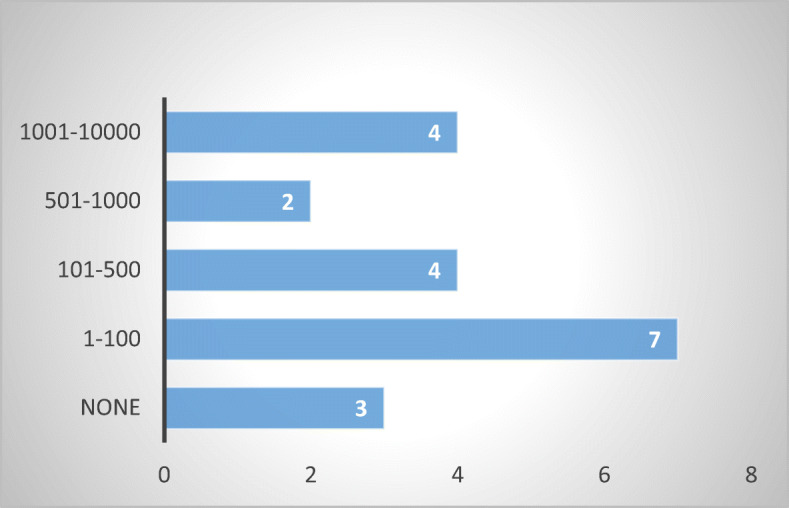
Number of complaints received—20 MS

**Fig. 7:**
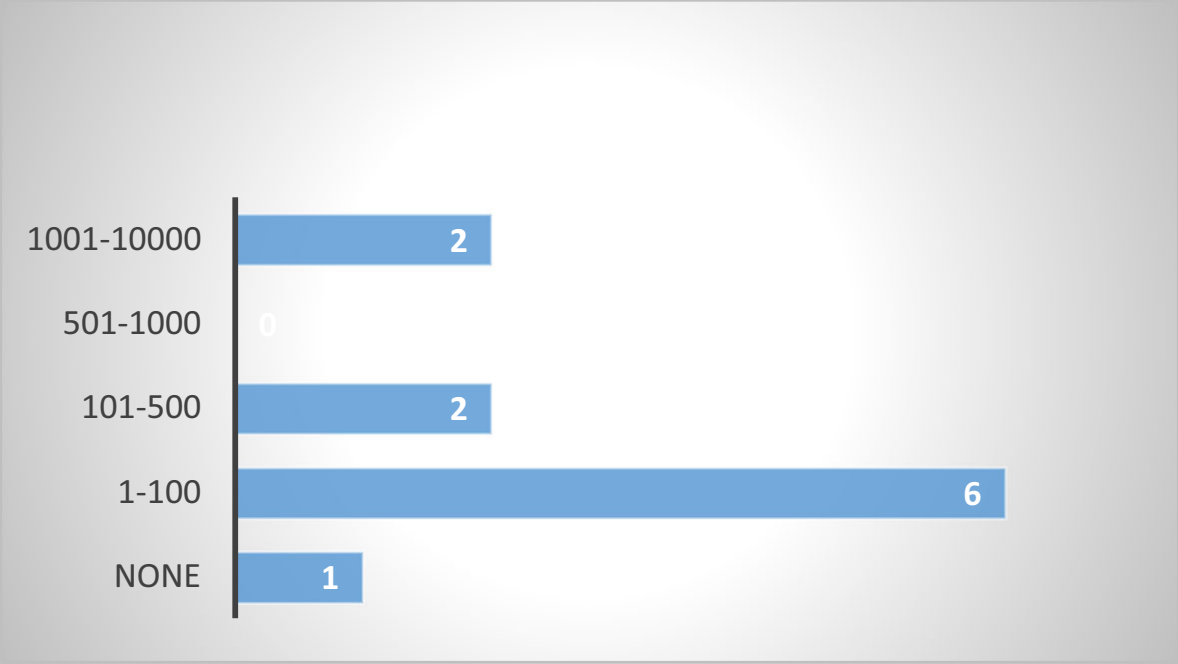
Number of complaints received by debt collection associations—10 MS

The number of complaints received in the past five years in each respondent MS varies, from less than 100 (or none) to 10,000, notwithstanding whether the complaints were addressed to competent state bodies or debt collection associations. While the numbers do not reflect the full picture or the exact magnitude of the issue, the results from national agencies and debt collection agencies are consistent with each other (Figs. 6 and 7).

**Fig. 8:**
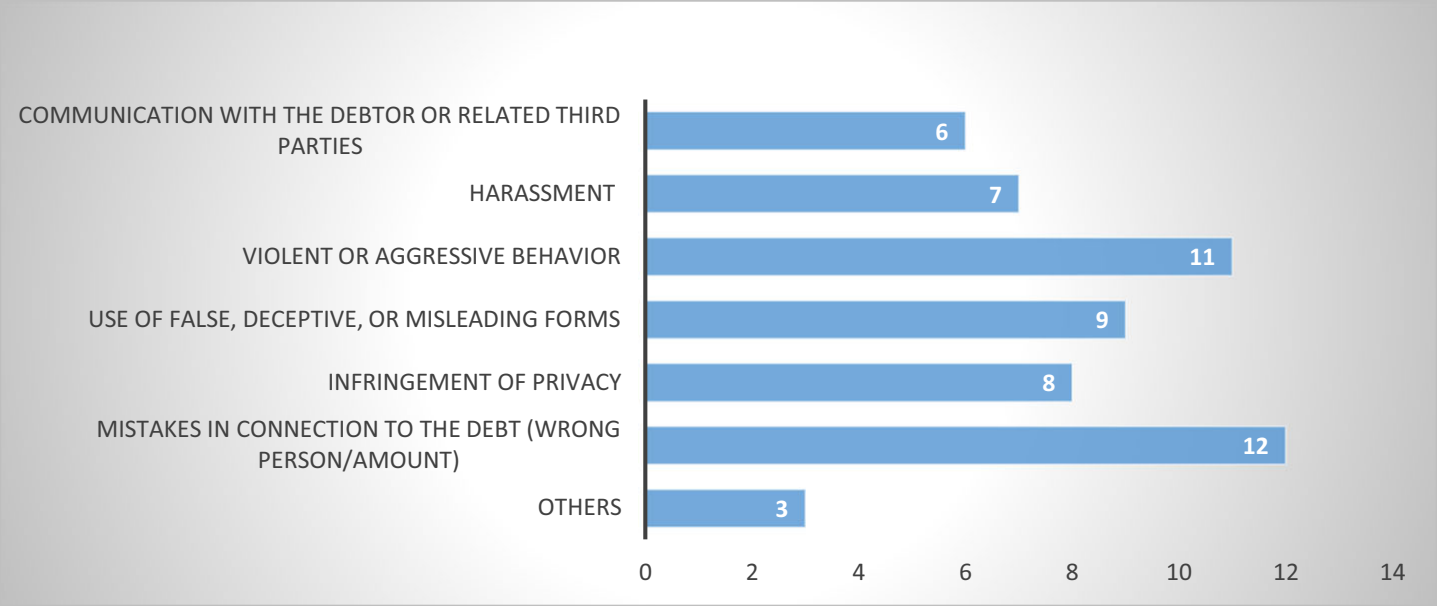
Types of abusive debt collection practices reported—15 MS

All types of abusive debt-collection practices are known in the EU and most MS experienced a wide spectrum of such practices. The answers obtained provide a sufficiently clear picture of the issues faced by EU consumer-debtors and corroborate parallel findings (Jérusalmy et al. 2020, pp. 33–37). The most frequent abusive practices involve debt-related “mistakes” concerning the amount and the validity of the debt or the identity of the debtor, followed by “violent or aggressive behaviour towards the debtor.” In addition, complaints concerned the “use of false or deceptive information,” “violations of privacy,” and “harassment” in connection to collection of the debt (Fig. 8).

One may notice that the survey neither provided definitions of listed practices nor requested them from respondents. The reason was twofold. On the one hand, I deemed confining respondents (especially those from MS with no sector-specific legislation in place) to a specific definition to be too limitative, as it may have precluded them from referring to the specificities and understandings of their own jurisdiction. On the other hand, asking respondents to engage in detailed explanations of their own understanding of said practices pertained the risk of low response rates. Thus, the categories were left to be construed as broad as possible, a manner which would have a minor impact on the overall result and left room for further research.

### Debt Validation, Suspension of Collection, and Added Charges

The survey also sought to ascertain whether the respondent MS provided consumer-debtors with the possibility to request validation of the debt (to challenge the existence, the amount or the validity of the debt) and, in close connection thereof, if the debt-collection efforts were stayed during the validation procedure (to avoid a wrongful collection). Although such possibility is usually provided by sector-specific legislation, the question was posed indiscriminately, which may have generated confusion and potentially imprecise answers, especially from the MS that do not regulate abusive non-judicial debt collection practices.

**Fig. 9:**
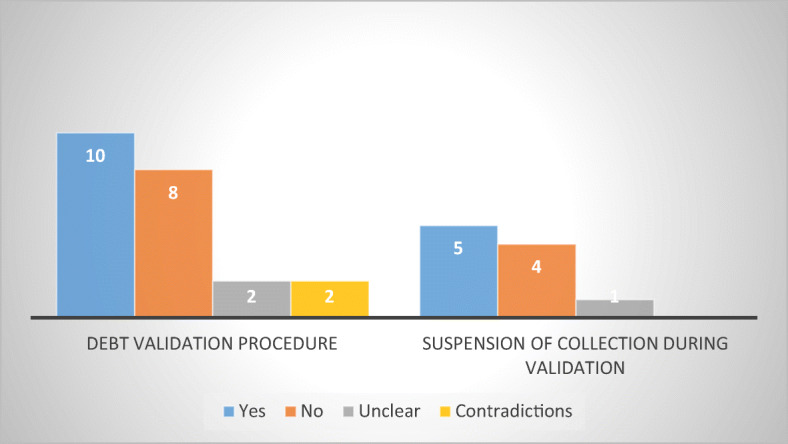
Debt validation vs. debt suspension in 22 MS

According to the answers received, in ten MS consumer-debtors can request validation of the debt. Among them, one can find five MS with sector-specific legislation (Denmark, Finland, Latvia, Romania, and Sweden) as well as five without (Croatia, Estonia, France, Ireland, and Slovenia). The fact that almost half of the respondent MS provide the consumer-debtor with a possibility to contest the debt, even in the absence of sector-specific obligations thereof, is a positive sign and an important mechanism for consumer protection (although the survey does not reveal how it functions) (Fig. 9).

However, the data collected indicates that although debt validation may be required, in half of the cases, collection efforts are not suspended throughout the validation procedure. Thus, from the MS that affirmed the existence of a validation mechanism, only five (Croatia, Denmark, Finland, Ireland, and Romania) provide for the suspension of collection. Surprisingly, Latvia and Sweden (from the regulated states), as well as Estonia, France, and Slovenia (from the unregulated ones) did not consider it necessary to stay collection efforts during validation, despite an obvious risk that the debt might be collected, even if challenged or erroneous.

**Fig. 10:**
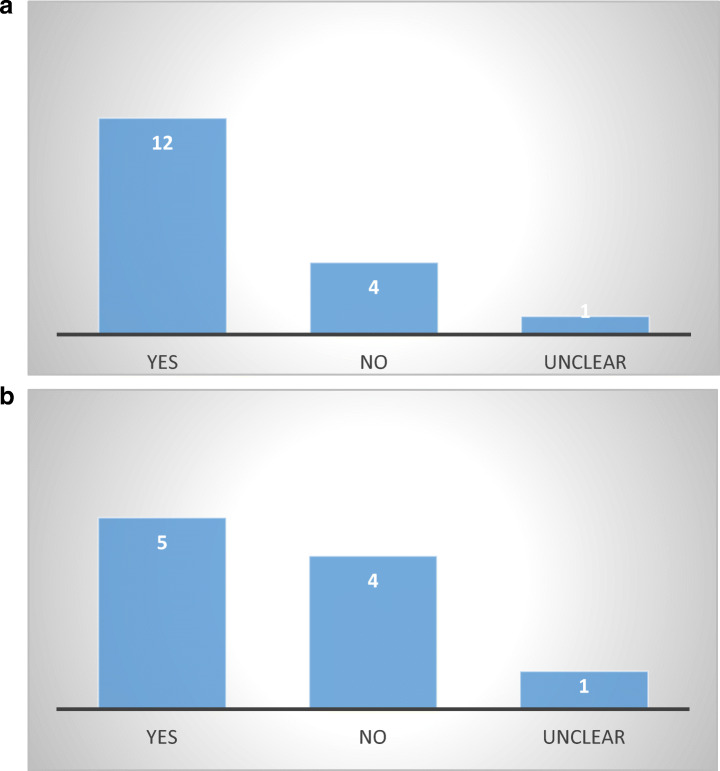
**a** Added charges according to consumer or supervisory agencies—17 MS. **b** Added charges according to debt collection associations—10 MS

Twelve out of seventeen respondents have indicated that debt collection costs can be added to the collected debt, which is a significant risk for the consumer-debtor who may experience even bigger difficulties in repaying as surcharges accrue in time. While some debt collection associations (France) see this as a means to pressure the debtor into faster repayment, the reality remains that such practice can only lead to an uncontrolled extraction of debtor’s funds (Fig. 10).

An additional issue regarding added charges stems from the contradictions noticed in the answers of consumer agencies and state bodies versus those received from debt collection associations from the same countries. For example, the Austrian consumer agency indicated that debt collection costs are not allowed, while the Austrian debt collection agency considers that they are. Conversely, the consumer agencies in Italy and Poland stated that collection charges are permitted, while the debt collection associations indicated the opposite. Such contradictions indicate that consumer-debtors may face uncertainties regarding their rights and obligations during non-judicial debt collection in countries with no sector-specific legislation.

### Remedial and Enforcement Options Available to Aggrieved Consumer-Debtors

Whether the MS has sector-specific legislation in place or not, it is important to determine what kind of remedies and enforcement mechanisms are available to consumer-debtors that are subjected to abusive non-judicial debt collection practices. The fact that debt-collection is unregulated does not necessarily mean that the consumer is completely unprotected: Administrative actions under general consumer protection laws, civil actions in tort, or criminal actions may still be present. The question is which ones are available and how effective are they (Jérusalmy et al. 2020, p. 38). It should be mentioned, once more, that the answers cover both states with and without sector-specific legislation.

**Fig. 11:**
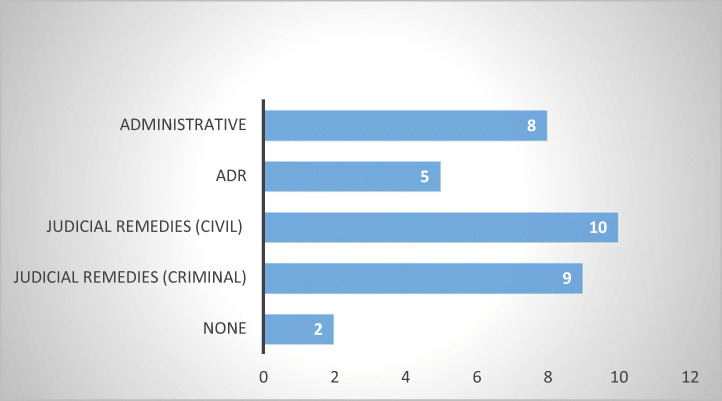
Types of remedies in 13 MS without sector-specific legislation

**Fig. 12:**
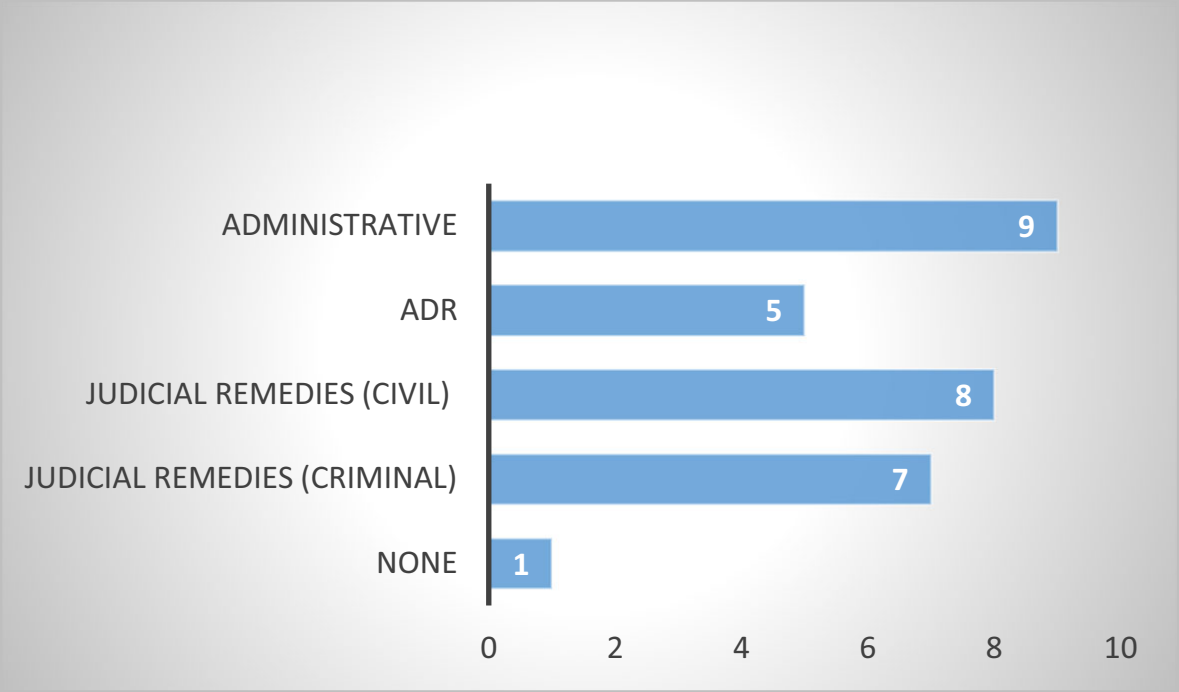
Types of remedies in 9 MS with sector-specific legislation

The survey revealed that civil and administrative remedies are the most common, being present in eighteen out of twenty-two MS, closely followed by administrative and criminal law remedies (present in seventeen, respectively sixteen out of twenty-two MS). Each deserve more attention (Figs. 11 and 12).

#### Civil Remedies

Eighteen of twenty-two respondent MS indicated the availability of a multitude of civil remedies: Belgium, Bulgaria, Czech Republic, Estonia, Finland, Germany,^24^ Greece, Ireland, Latvia, Lithuania, Luxembourg, the Netherlands, Poland, Romania, Slovakia, Slovenia, Spain, and Sweden.

**Fig. 13:**
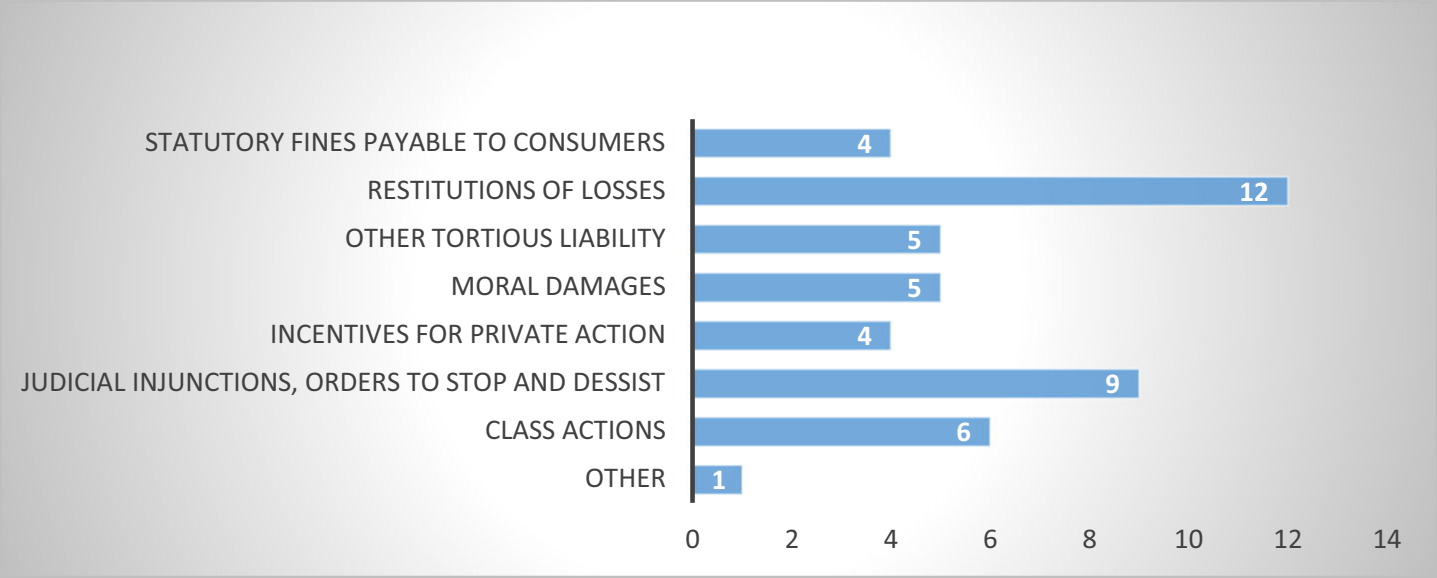
Civil judicial remedies—16 MS

The most common appears to be the restitution of losses suffered by the aggrieved consumer-debtor due to abusive debt collection practices, which was reported in twelve respondent MS (Belgium, Bulgaria, Estonia, Germany, Greece, Latvia, Netherlands, Poland, Romania, Slovakia, Slovenia, and Spain). Surprisingly, however, only five respondents indicated other tort remedies (Finland, Germany, Greece, Poland and Romania), although in my assumption, in abusive debt collection cases not arising from sector-specific legislation, restitution of losses incurred by the consumer-debtor can arise solely from an action in tort. This inadvertence, may be stemming from the respondents’ knowledge of civil law and civil procedure and the likelihood is that all MS reporting the restitution of losses have tortious actions in place (Fig. 13).

Nine respondents indicated the presence of judicial injunctions (Czech Republic, Estonia, Finland, Germany, Ireland, Netherlands, Poland, Slovakia, and Spain) and five reported the possibility to obtain moral damages, for wounded feelings, emotional distress, loss of reputation (Germany, Latvia, Poland, Romania, and Slovenia). Class actions are available in six of the respondent MS (Czech Republic, Finland, Germany, Netherlands, Poland, and Slovakia). Finally, yet importantly, statutory fines payable to the aggrieved consumer-debtor are reported in four of the respondent MS (Finland, Germany, Poland, and Sweden).

From a procedural standpoint, of relevance is the fact that only four respondents indicated the existence of incentives offered to consumers to take private action (Estonia, Germany, Poland, and Slovakia), although it is not clear what these incentives consist of.

From the above, it becomes apparent that there is a wide mosaic of civil remedies available to consumer-debtors subjected to abusive debt collection practices. Where a sector-specific legislation is present, these remedies stem from the applicable *lex specialis*, while in countries with no such legislation in place, these remedies have a general status, available for any kind of civil wrongdoing suffered.

**Fig. 14:**
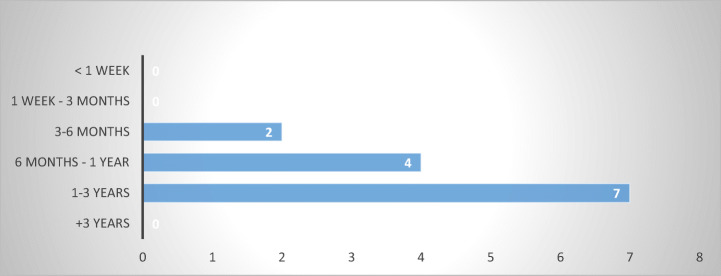
Duration of civil proceedings—13 MS

The question is how efficient are these civil remedies, and how long would it take for a consumer-debtor to pursue civil proceedings against an abusive debt-collector? Based on the answers obtained, civil procedures are rather lengthy, with seven out of thirteen MS reporting a duration between one and three years (Estonia, Finland, Luxembourg, Netherlands, Poland, Slovakia, and Slovenia) (Fig. 14).

This means that in order to gain access to the civil remedies available, consumer-debtors aggrieved by abusive debt-collection practices would have to go through a lengthy and, thus, (potentially) expensive procedure, which might exceed the actual loss incurred or the gain, envisioned. In the absence of incentives for private action (such as exemption from judicial taxes and/or judicial fees, reversed burden of proof, expedite procedures) and specific remedies (statutory fines payable independent of actual losses incurred), the length and the expenses associated with a civil trial will act as a deterrent to consumer action (Caponi and Novak 2019, p. 65) against abusive debt collectors, especially in countries lacking a sector-specific legislation. Thus, based on the findings of the survey, one can venture that their efficiency as an alternative to regulation of abusive debt collection is going to be rather low.

#### Administrative Remedies

Besides MS that have a sector-specific legislation (Belgium, Denmark, Finland, Germany, Greece, Latvia, Netherlands, Romania and Sweden), administrative remedies were reported also by Czech Republic, Estonia, Ireland, Italy, Poland, Slovakia, Slovenia, and Spain. However, in the latter’s case, it is not clear what the origin of administrative remedies is in the absence of specifically designed rules to tackle abusive debt collection.

Since administrative remedies vary, the survey also sought to determine what types of remedies are present in each respondent MS. The expectation was that due to differences in legal systems and approaches, the administrative remedies vary significantly from one MS to another.

**Fig. 15:**
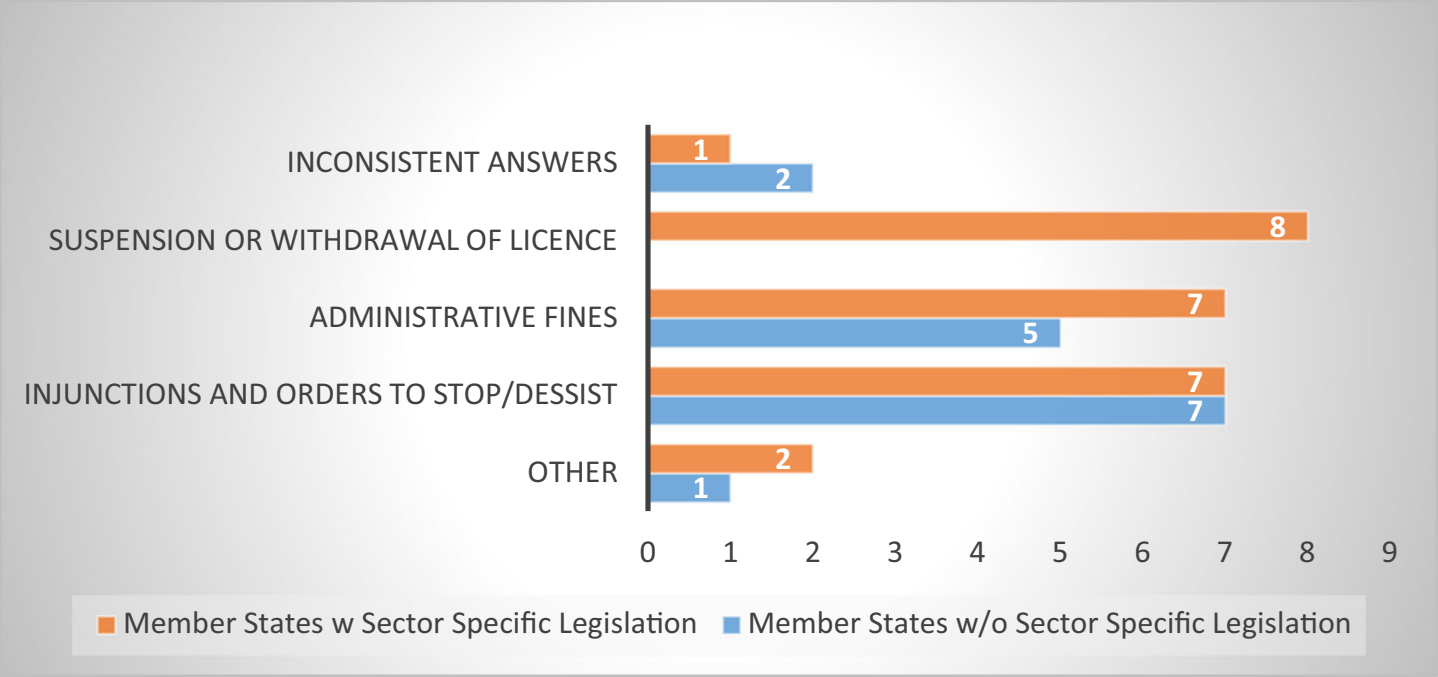
Types of administrative remedies (17 MS)

The results showed that MS with sector-specific legislation combine several types of administrative remedies. For instance, Denmark, Germany, Greece, and Latvia use suspension of licence, fines, and injunctions. Finland and Sweden resort to injunctions and suspension of licence, Belgium and Romania combine administrative fines with suspension of licence, while Netherlands resorts to injunctions and administrative fines (Fig. 15).

From the group with no sector-specific legislation in place, Slovakia appears to be the MS with most administrative remedies in place: injunctions, fines, and others, followed by Estonia, Italy, and Poland, who use injunctions and fines. The remaining respondent MS have indicated only one type of administrative remedy available: Czech Republic, Ireland, and Spain resort to injunctions, while Slovenia relies on fines.

There are also contradictions. The respondents from the Netherlands, Czech Republic, and Slovakia have previously indicated that no licence to operate is needed; however, suspension of licence was listed among the administrative remedies available. The contradiction may stem either from an erroneous response to one of the questions or from an erroneous understanding of the questions. Due to the incumbent contradictions, their answers were deemed inconsistent and counted separately.

One should note that the eight MS that indicated suspension of licence as an administrative remedy belong to the group of MS that regulate abusive debt-collection practices via tailor-made legislation. While this reflects the importance of licencing as a first line of defence against businesses that engage in unscrupulous practices, it also suggests that suspension might function better when linked with standards of conduct and specifically banned practices, which enable the supervisory bodies to establish if a violation has occurred.

Twelve of the seventeen respondent MS indicated fines as an administrative remedy. To be efficient, these should reach a level that would deter the penalized debt-collectors from engaging in abusive practices again, without being punitive or disproportionate.^25^ Thus, the survey sought to determine the level of administrative fines applicable in the respondent MS. Ten respondents indicated fine levels of over 10,000 euro (Belgium, Estonia, Germany, Greece, Italy, Latvia, the Netherlands, Poland, Romania, and Slovakia), one indicated amounts between 1000 and 5000 euro (Slovenia), while another reported levels below 100 euro (Denmark). Although the results seem to indicate that the MS that resort to fines tend to set them towards a higher level, their success is hard to evaluate. Given that debt collection agencies have an annual turnover in millions or tens and hundreds of millions of euro,^26^ the level of fines may not be deterrent enough. A more adequate solution would be to link fines to the annual turnover of the company (using a system similar to competition or GDPR rules, as the one adopted by the recent Enforcement Directive 2019). From the respondent MS, only Latvia uses a fine up to 10% of the net turnover of the last financial year for the unfair commercial practice, however capped at 100,000 euro.

Another matter of importance is the duration of administrative proceedings and the survey sought to determine whether they address in a swift and compelling manner the issue of abusive debt collection practices.

**Fig. 16:**
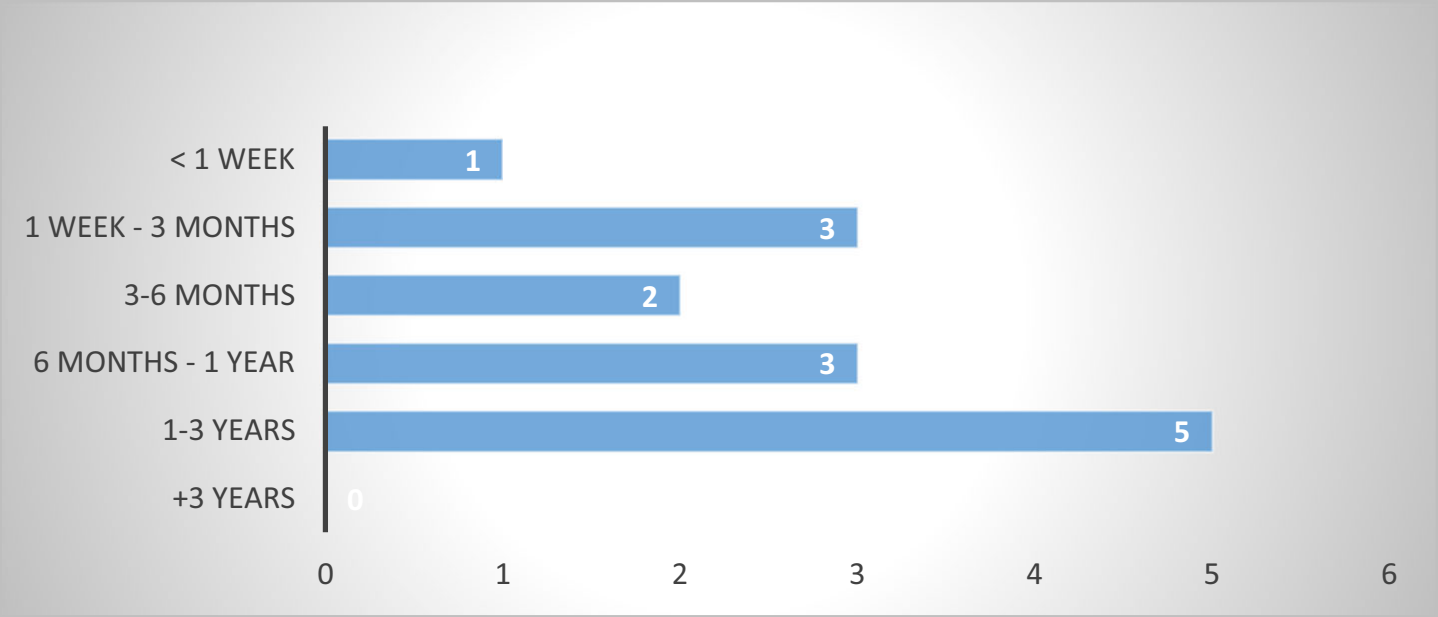
Duration of administrative proceedings—14 MS

The results show diversity in duration of administrative proceedings, the most common ranging from one to three years (Estonia, Germany, Ireland, the Netherlands, and Slovakia), from six months to one year (Finland, Italy and Poland), and from one week to three months (Czech Republic, Slovenia, and Sweden). At the extreme, one respondent specified a duration of less than a week (Denmark). Several respondents from Germany also indicated a duration of less than a week; however, in this instance, preference was given to the longest period reported. The results are largely consistent with the 2018 EU Justice Scoreboard (European Commission 2018, pp. 11–13) (Fig. 16).

While data is absent from several MS, the partial results reveal discrepancies in duration of administrative proceedings, which has an automatic impact on their efficiency across the internal market. Long durations do not seem to be associated with the level of development of the state and the maturity of its institutions, as Germany and the Netherlands, for instance, reported similar results to those of Estonia, Ireland, or Slovakia. The explanations lie elsewhere. For the moment, suffices to say that administrative proceedings appear not to provide an equally swift respite to consumer-debtors across the union.

#### Criminal Remedies

**Fig. 17:**
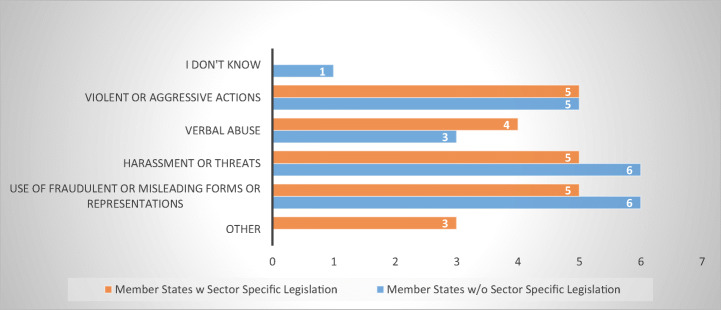
Types of criminalized offences that could be used by consumer-debtors—15 MS

Since abusive debt collection practices could amount also to criminal offences, especially where physical violence, threats, harassment, and verbal abuse are involved, the survey sought to establish whether the criminal remedies available in the respondent MS could provide an efficient alternative to sector-specific legislation (be it present or not) (Fig. 17).

**Fig. 18:**
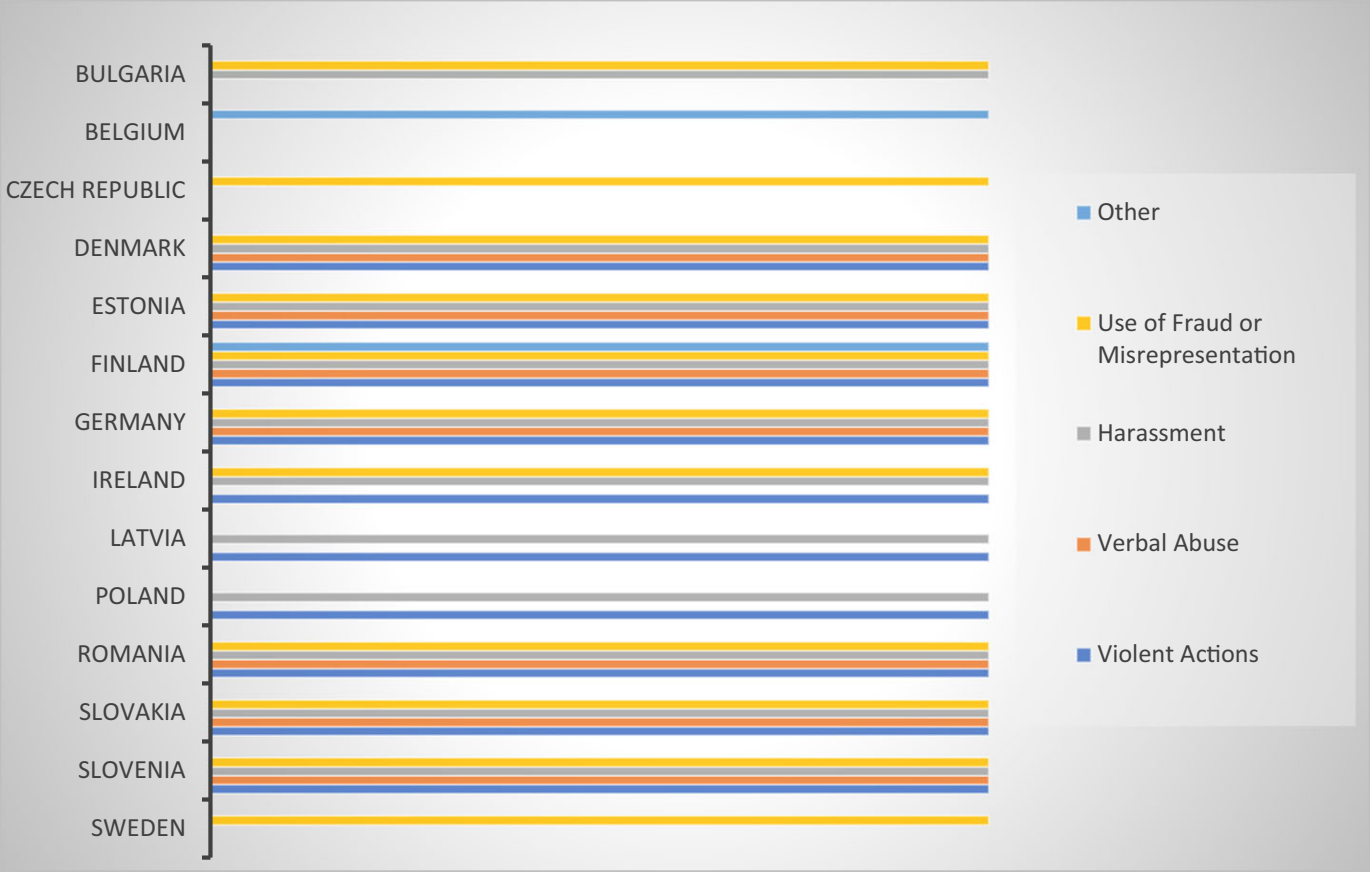
Criminalized offences in 14 MS

The results revealed that fifteen respondent MS (Belgium, Bulgaria, Czech Republic, Denmark, Estonia, Finland, Germany, Ireland, Latvia, Luxembourg, Poland, Romania, Slovakia, Slovenia, and Sweden) criminalize several abusive practices, such as use of fraudulent or misrepresentative acts, harassment, and the use of violence or verbal abuse. The most frequent appeared to be the use of fraudulent means and misrepresentations as well as harassment and threats, each reported by eleven of the respondents, five belonging to MS with sector-specific legislation and six to MS without (Fig. 18).

Violent actions against the consumer-debtor (or his family) are criminalized in at least ten of the responding MS. This result comes out as surprising, given that one cannot expect violent actions not to be criminalized at all. Should one understand that consumer-debtors are not insulated from violent actions? The response is unlikely to be positive and, notwithstanding whether it stems from the lack of knowledge or understanding of the respondents, it should be treated with suspicion.

**Fig. 19:**
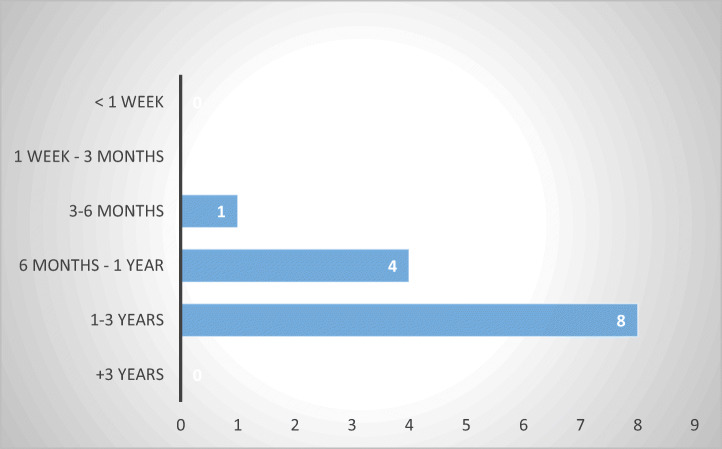
Duration of criminal proceedings—13 MS

As in the case of administrative and civil remedies, duration of criminal proceedings is a relevant factor in determining whether criminal remedies constitute an adequate alternative to sector-specific legislation protections (Fig. 19).

Similar to civil remedies, most respondents (eight out of thirteen) indicated that criminal proceedings take between one and three years (Belgium, Bulgaria, Estonia, Finland, Ireland, Luxembourg, Poland, and Slovakia), while others reported procedures between six months and one year (Czech Republic, Germany, Slovenia, and Sweden) or three to six months (Denmark).

The length of criminal procedures and the extent to which abusive debt collection practices are perceived to constitute criminal offences vary among MS, just like in the case of civil or administrative proceedings, thus, exposing consumer-debtors to the vagaries of their home legal systems. Given that the most common duration identified stretches between one and three years, the likelihood that consumers will find criminal proceedings of use against abusive debt collection practices is rather low.

#### Alternative Dispute Resolution Mechanisms

**Fig. 20:**
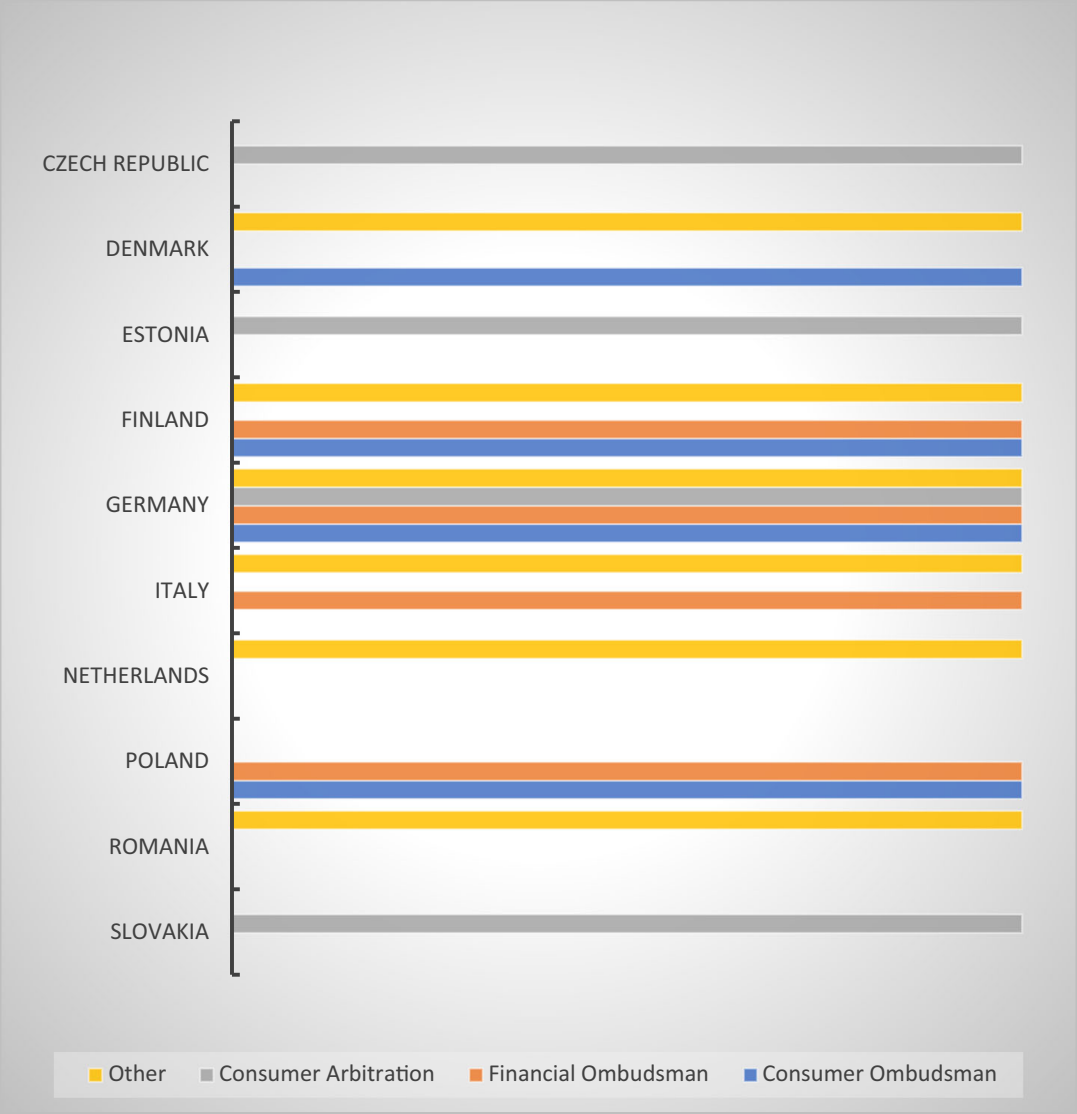
Types of ADR mechanisms available to debtors in regard to abusive debt collection in 10 MS

MS provide aggrieved consumers with access to Alternative Dispute Resolution (ADR) mechanisms.^27^ These bodies are generally able to handle complaints swiftly, either free or for a nominal fee, which makes them more attractive. The survey sought to determine in how many of the respondent MS consumers have access to ADR mechanisms, what those mechanisms are, and how long it averagely takes them to render a resolution. In addition, I inquired whether their decisions are binding on the debt-collectors (meaning that they can be immediately enforced) and whether the decisions are made available to the public in any form (which would have the benefit of providing all stakeholders with increased legal certainty and transparency) (Fig. 20).

**Fig. 21:**
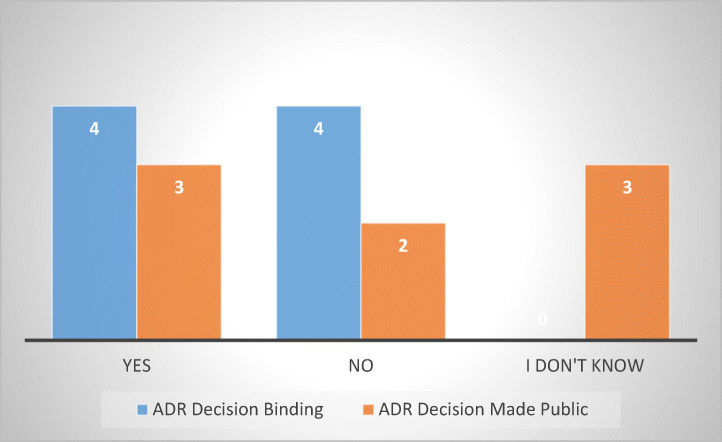
ADR decisions—binding and public—8 MS

As it is apparent from the graph above, all options are equally distributed. The Financial Ombudsman is available in four of the respondent MS (Finland, Germany, Italy, and Poland) just like the Consumer Ombudsman (Denmark, Finland, Germany, and Poland) or consumer arbitration forums (Czech Republic, Estonia, Germany, and Slovakia). Other ADR bodies are also present: the Consumer Dispute Board (Finland), the Danish Bar and Law Society (Denmark), Antitrust Committees (Italy) or mediation (Romania). Once again, the responses reveal a relatively wide array of ADR mechanisms available, some of the MS even providing consumers with several options (Finland, Germany, Italy or Poland), although in the absence of sector-specific legislation, it is uncertain whether a consumer-debtor aggrieved by abusive debt collection practices could bring a complaint to any of them (Fig. 21).

Four respondent MS indicated that decisions of ADR bodies do not have a binding effect (Czech Republic, Estonia, Finland, and Germany). As already stated, this may undermine the effectiveness of ADR mechanisms and diminish consumers’ trust in their role and purpose. That is because in many cases, the value of the claim is low, and the consumers will not be inclined to pursue it further in court, an aspect which has been confirmed by other empirical studies (Caponi and Nowak 2019, pp. 65–68).

Thus, in MS with no binding effect of ADR decisions, it is debatable whether they can provide an adequate alternative to civil or criminal remedies. The result is consistent with other empirical studies, although the lack of binding effect has been also considered to have a positive effect in ensuring that areas of consumer protection law are not shielded from the civil judiciaries (Hess and Taelman 2019, p. 129; Hodges 2019, pp. 182–184, 190–191).

Of relevance is also the issue of whether the decisions are made public, which should increase awareness and legal certainty for all stakeholders. Half of the respondents indicated that ADR decisions are generally accessible to the public, online (Estonia, Finland, and Italy). Two respondents (Germany and Slovakia) indicated a negative answer, while three (Czech Republic, Denmark, and Poland) stated that they do not know. Once again, the lack of knowledge displayed by some respondents (most of them state bodies or consumer associations) is staggering, and one may question the adequacy and professionalism of their service. What is certain is that in the absence of public case law, it will be difficult, especially for consumers, to discern the practice and approach of the ADR body with respect to the issue at hand, thus undermining transparency and consumer confidence.

**Fig. 22:**
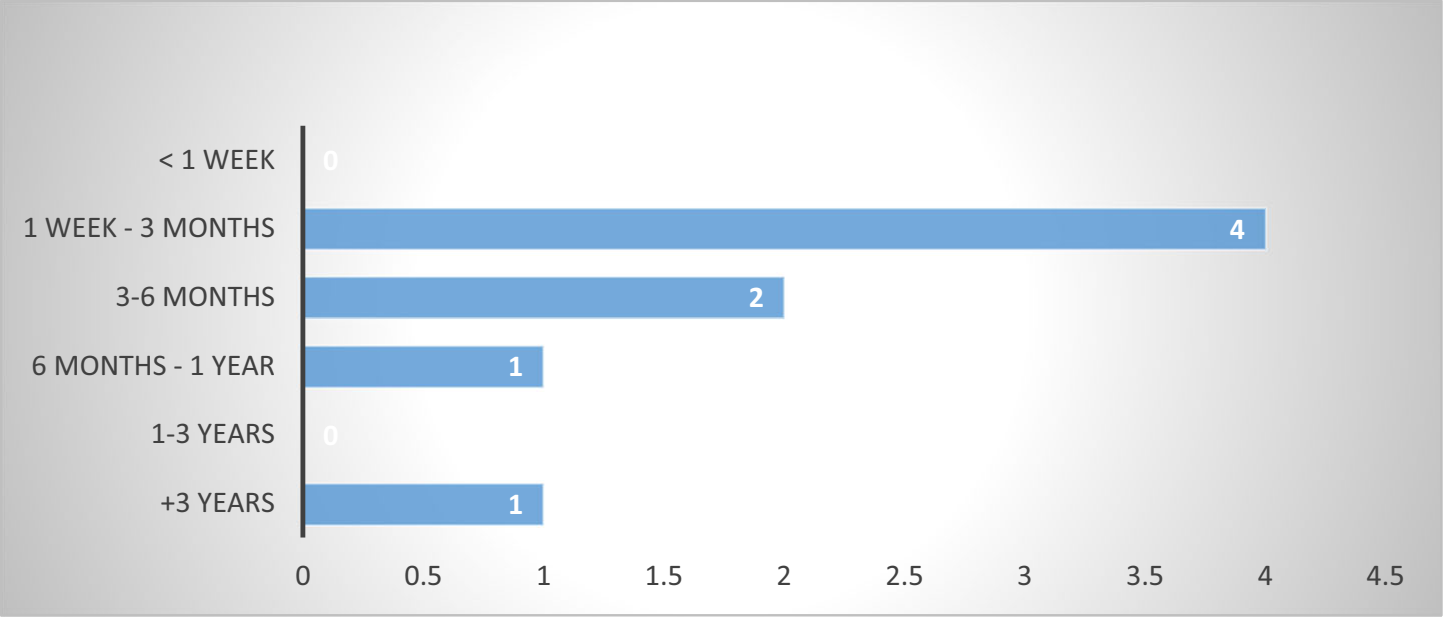
Duration of ADR proceedings—8 MS

Finally, the survey revealed that ADR mechanisms deliver a swifter outcome than civil or criminal proceedings, with six of the respondents indicating a duration between one week and six months. This result is also consistent with other empirical findings according to which consumer ADR tends to be quicker, less costly, and more accessible compared to court proceedings (Hess and Taelman 2019, p. 129) (Fig. 22).

From this perspective, ADR mechanisms appear to be the best-suited proceedings for bringing a claim against an abusive debt-collector, provided the ADR body is empowered to hear claims in this regard. However, the survey was unable to discern their functionality in a cross-border setting.

### The Role of Codes of Conduct and Industry Self-Regulation

I have already mentioned the limited role-played by Codes of Conduct in addressing abusive debt collection practices. Since a significant number of respondents either indicated their absence, or were unaware of their existence suggests that there is an uneven playing field (Jérusalmy et al. 2020, p. 28) and that Codes of Conduct cannot replace sector-specific legislation in the assessment and handling of complaints. Their role is complementary, and it is either a matter of regulatory intervention (where they are imposed by regulators) or one of self-governance (where adopted at a company or industry level).

**Fig. 23:**
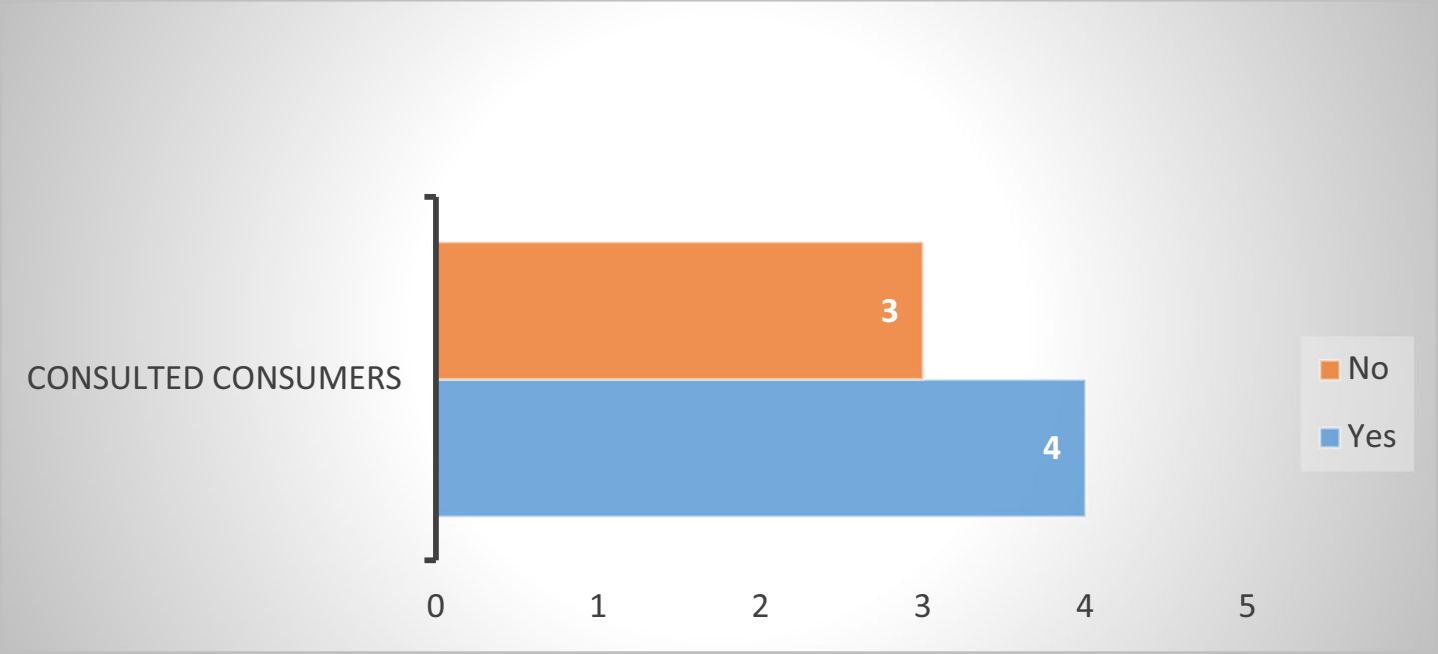
Consulted consumers in designing Codes of Conduct for debt collectors—7 MS

This is supported by evidence stemming from debt collection associations in ten EU MS (Fig. 23). Seven of them indicated that Codes of Conduct are present, yet in only four (Belgium, France, Italy, and Sweden) consumer organizations were consulted in their drafting.

**Fig. 24:**
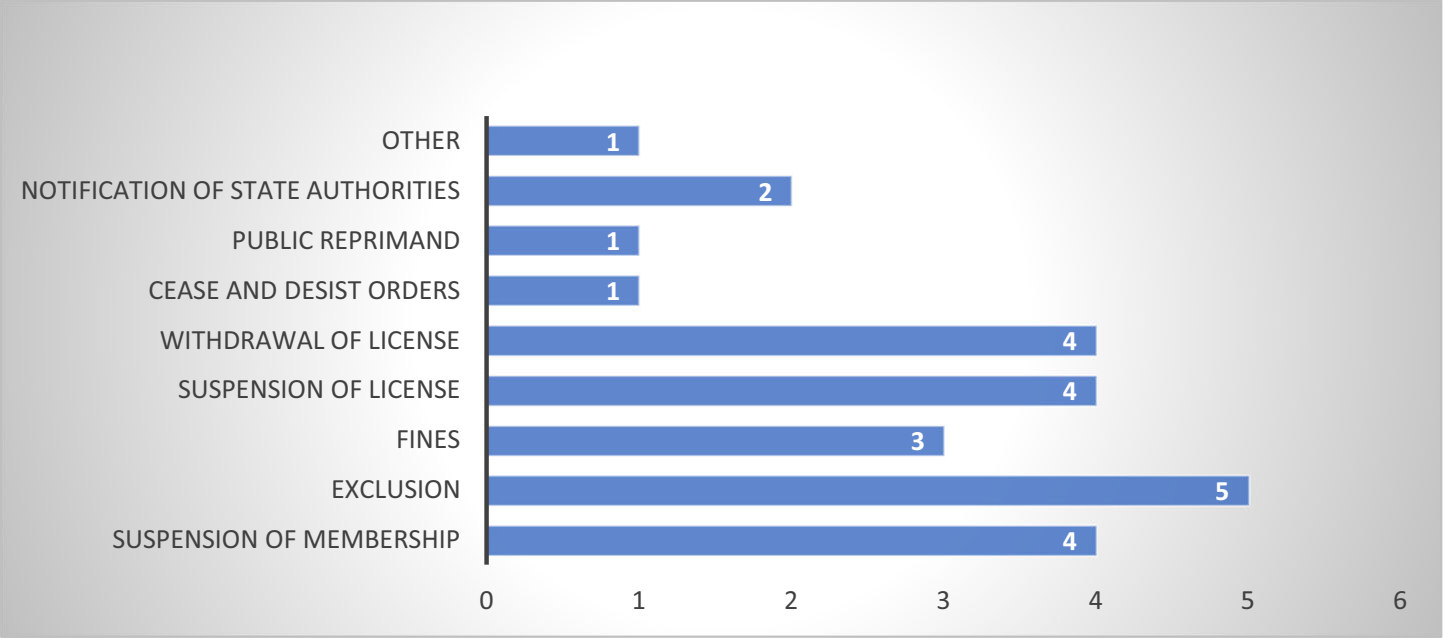
Types of sanctions present in Codes of Conduct—7 MS

Based on the data provided by the seven respondent debt collection associations that indicated the presence of a Code of Conduct, all of them ban abusive debt collection practices and provide for sanctions for non-compliance (Fig. 24).

The most common appears to be exclusion from the debt collection association. However, in the absence of a combined state action or a condition to become a member of a debt collection association to practice, the exclusion does not affect the activity or the possibility of the wrongdoer to continue operation. This confirms parallel results according to which, in half of the countries where Codes of Conduct are used, they are not effective (Jérusalmy et al. 2020, pp. 38–39). In contrast, notification of public authorities or public reprimands of non-compliant members is reported solely by two (France and Sweden), respectively one (Sweden) of the respondents. Withdrawal or suspension of licence may occur in cases where a regulatory authority imposed the Code of Conduct, although based on the information received via the survey it not possible to state this with absolute certainty.

**Fig. 25:**
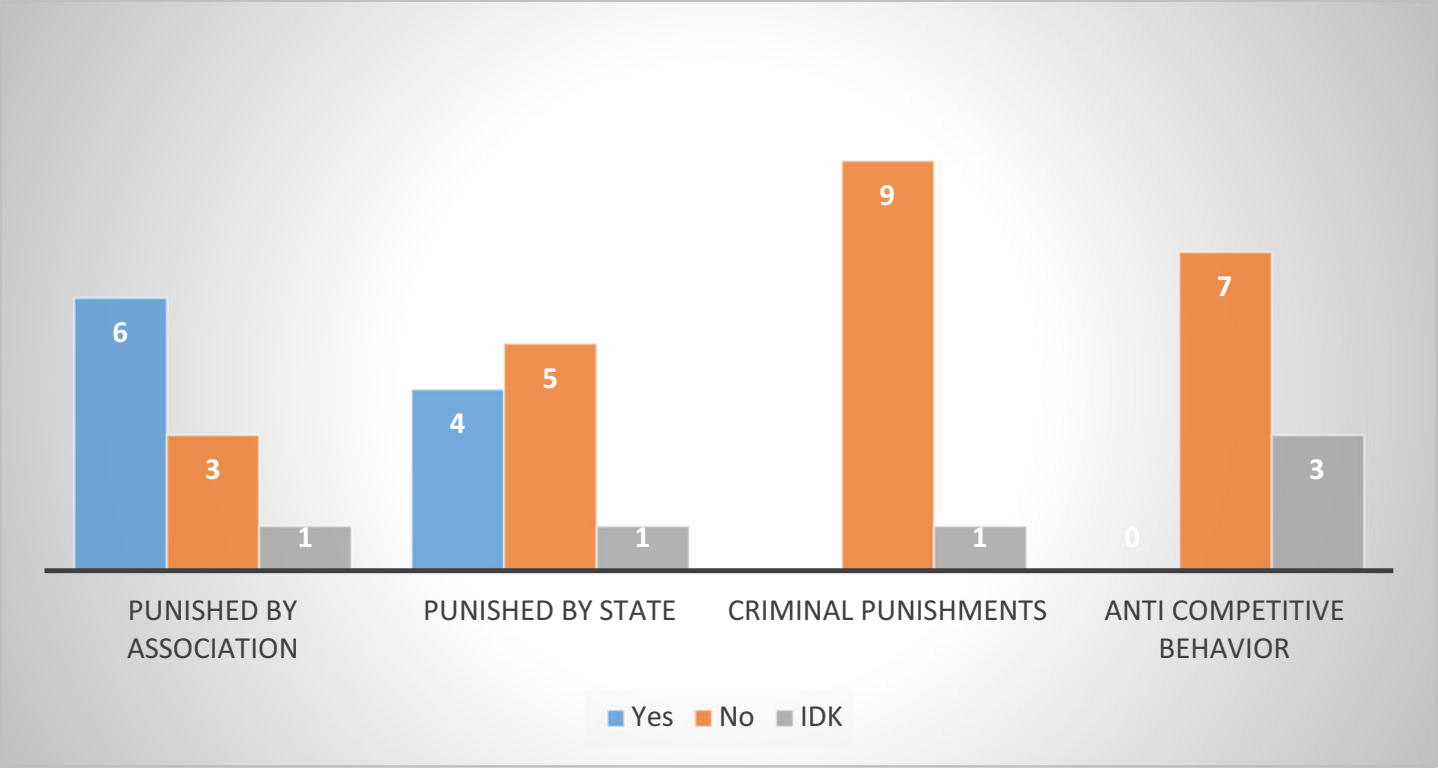
Penalized members of debt collection associations—10 MS

I also tried to ascertain whether members of respondent debt collection associations have been punished for engaging in abusive debt collection practices. If so, by whom and under what framework? (Fig. 25)

Six associations (Austria, Belgium, Czech Republic, Germany, Poland, and Sweden), indicated that they had members that were penalized for engaging in such practices. Four respondents (Belgium, France, Poland, and Sweden) stated that they had members who were penalized by the state authorities. None of the respondents had, to the best of their knowledge, any members held accountable under criminal law or under fair competition rules for engaging in abusive debt collection, which would suggest that these traditional remedies have a low efficiency in practice (at least in the respondent MS).

### The Open-End Question: Is There a Need for Regulation of Abusive Debt Collection Practices in the European Union?

The survey’s ultimate purpose was to establish if the current national regulations of abusive debt collection practices suffice or there is room for improvement at either MS or EU level. The quantitative data provided a clearer image of the level and standards of protection that is existent in the EU against abusive debt collection and enabled readers to form their own conclusions. The open-end question, however, served to obtain the standpoint of the respondents to the survey in their stakeholder capacity (regulators, supervisors, or subjects) vis-à-vis debt collection regulation. Results are largely consistent with parallel findings (Jérusalmy et al. 2020, pp. 39–40).

The stakeholders who responded to the open-end question agreed that abusive debt-collection is a problem for both consumers and ethical businesses, and thus, needs to be addressed and regulated, while some suggested areas that deserve attention in their national context towards improving fair and honest treatments of consumers. I will sum up these answers here before drawing my own conclusions.

#### Regulators, Consumer Associations, and Supervisory Bodies

There were concerns regarding the lack of rules for licencing debt collectors given that “anyone can start a debt collection agency” and not all of them chose to become members of the debt collectors association, where they would have to comply with the Code of Conduct (Netherlands). Thus, licencing of debt collectors, membership in the national association, and compliance with a Code of Conduct should be mandatory (Estonia, Netherlands, and Poland) and a duty of care should be imposed (Netherlands).

Others emphasized the need for a “clear legal framework for the activity of debt-collectors” (Estonia and Germany) and for a “black list that should clearly define and detail the debt collection procedure,” together with the need for improvement in the enforcement of the UCPD 2005, given that “many unlawful practices of debt-collectors go unpunished” (Germany).

An obligation to provide the consumer with clear information and evidence concerning the debt and the added costs (Germany, Estonia, and Slovakia), as well as the need for clear communication rules (including related third parties, such as underage children) and limits to debt-collection related surcharges were also suggested (Estonia and Slovakia).

One opined, that “it would be great if this legislation might be applicable all over Europe” and suggested that legislators should ensure that no more confusion between judicial and non-judicial debt collection is possible (which allowed debt collectors to impersonate bailiffs) (Belgium). Finally, one respondent stated that its MS has a “relatively good legal regulation” and does not propose any improvements (Latvia).

#### Debt Collection Associations

The debt collection associations generally acquiesced to the position that abusive practices and non-competitive behaviours “are not acceptable” and should be treated “seriously” (Romania), that “special regulation of debt collection needs to be introduced” (Bulgaria, Czech Republic) and that such regulation “would constitute a positive development” (Bulgaria). Along the same lines, it was suggested to turn (the future) Code of Conduct adopted by the industry into law (Germany), thus making it mandatory for all debt collectors. However, sector-specific legislation should balance consumer, creditor, and industry interests (Bulgaria).

In addition, some stressed the need to distinguish between ethical and non-ethical debt collectors, to sanction those who affect the image of the industry with their behaviour (Bulgaria, Czech Republic, and Romania), to enforce minimal qualifications for all debt collectors (Czech Republic), and limit additional charges levied on the consumer-debtor by making them “reasonable” (Germany).

There were also opinions that the current general regulation of consumer protection suffices and no sector-specific rules are necessary, given that “the debt collection market is an important part of the financial market, which is currently tightly regulated by the legislator and supervised by authority bodies” (Poland). At the same time, one of the two French associations suggested implementation of additional penalties against consumer-debtors in default, which, in its opinion, would decrease the cost of debt collection for creditors and “smooth the debt collection process.”

## Discussion

The research sought to establish, in a comprehensive manner, if and how abusive debt collection practices are regulated in the respondent EU MS. In subsidiary, it aimed to provide an updated insight into the existence of a licencing regime for debt collectors, the transboundary dimension of debt collection and its implications for the single credit servicing market, the types of encountered abusive practices and the viability of various remedies available to consumer-debtors. Ultimately, it sought to answer the question whether regulation of abusive debt collection practices is needed, at MS or EU level.

Based on the answers received, the regulation of abusive debt-collection practices is not addressed equally throughout the MS. The empirical data revealed that almost two thirds of the EU consumer-debtors does not enjoy tailored-made protection from abusive debt collection practices. Moreover, the study suggests that the UCPD 2005 may not be a viable alternative to sector-specific legislation (be it national or EU law) for two main reasons. First, only about a half of MS claimed to resort to its provisions. Second, the UCPD 2005 is largely employed by MS that already have sector-specific legislation concerning abusive debt collection. Therefore, it appears to serve a complementary purpose and is not the primary source of regulation. It is difficult to speculate an answer as to why the UCPD 2005 is not used more widely across the EU. It may be, as shown elsewhere (Stănescu 2020), that the UCPD 2005 is simply unfit to assume a greater role in tackling abusive debt collection.

The data revealed that most debt collectors operate in a legal uncertainty, taking advantage not only of the absence of sector-specific legislation, but also of the lack of licencing and minimum occupational standards. In many MS anyone can be a debt-collector. Out of the twenty-two respondent MS at last, ten do not require debt collectors to hold a licence to operate (Bulgaria, Croatia, Czech Republic, Estonia, Ireland, Luxembourg, Lithuania, the Netherlands, Poland, or Slovenia). This creates significant risks for all stakeholders as they may be dealing with unscrupulous individuals or businesses, with little regard for ethics and professional deontology. Moreover, it creates potential for unfair competition and encourages a race to the bottom between debt collectors, because there is little incentive or pressure to behave responsibly.

Licencing of debt buyers and debt collectors is a matter of interest also in connection to the NPLD Proposal. According to the NPLD’s Impact Assessment^28^ the introduction of a harmonized, pan-EU authorization, would reduce the costs associated with the procedure and compliance, and would benefit firms acting in different jurisdictions, by providing them with an EU passport that eliminates the need of authorization in each jurisdiction.^29^ Fifty-eight percent of the consulted stakeholders were in favour of a harmonized licencing regime (Impact Assessment, p. 75).

The idea that currently debt-collectors face hurdles in conducting their activity cross-border appears unsupported by the data. On the one hand, almost half of the respondent MS do not require any licence for debt collection, while, on the other hand, following the European Cout of Justice’s decision in *EU Commission v. Italy*,^30^ MS cannot impose burdensome licencing procedures on foreign debt collectors without breaching one of the EU fundamental freedoms. On the contrary, it might bring debt-collectors under supervision no matter where they choose to conduct their activities. Seen from this perspective, a pan-EU, harmonized licencing requirement for debt collectors will prove beneficial to all parties involved and will reduce the likelihood of engaging in unscrupulous cross-border debt collection. Nevertheless, in order to be truly functional, the harmonization of licencing needs to be seconded by a harmonized minimum standard of fair behaviour, as those advanced in the amendments to the NPLD Proposal (CEMA 2019). The need to provide for minimum standards of consumer protection in civil proceedings in order to improve consumer access to justice, and increase legal certainty, has also been emphasized in similar pan-EU empirical studies (Hess and Law 2019, p. 5).

The survey confirmed the hypothesis that abusive debt collection practices are widely known and spread in the EU. At least seventeen out of twenty respondent MS reported complaints thereof. The figures should not be construed in the sense that such practices are absent from the remaining MS (from the respondents, only three indicated no complaints received, while seven MS provided no data). The fact that in over half of the MS that reported complaints these concerned the actions of foreign debt-collectors highlights the cross-border dimension of the debt collection industry in the EU. Thus, any solution should acknowledge it and have a transnational dimension as well, aiming at achieving harmonization.

The likelihood of cross-border debt-collection is expected to grow. According to the NPLD Proposal’s Impact Assessment, the pan-EU licence and the low compliance costs associated with it will encourage firms acting in different jurisdictions and enable all debt-collectors to realize scale economies by tapping markets across the EU (Impact Assessment, p. 76).

However, this is not necessarily good news for local companies and consumers. The generalization of cross-border debt collection will increase competition among debt collectors, which might lead to increased size and market concentration, with the corollary of smaller players being eliminated or absorbed by larger ones. Increased competition in the absence of adequate regulation of debt collection practices will also increase the likelihood of abuse, the victims of which will be consumer-debtors, an aspect underlined also by a parallel study (Jérusalmy et al. 2020, p. 40).

Consumers are expected to face welfare losses, especially if the counterpart is authorized in a different MS.^31^ Thus, if the NPLD Proposal will come into being, one should expect cross-border debt collection to become the norm, at the expense of consumers who reside in MS that lack sector-specific protections and adequate mechanisms to protect them against abusive practices. According to the NPLD Proposal, interest in consumer-welfare is rather low, only 16% of the stakeholders indicating the need to improve debtor protection. This accentuates the need for regulatory intervention and the adoption of harmonized standards of behaviour.

Based on the collected data, the spectrum of abusive practices is quite large the most frequent of which concern “errors” regarding the debt followed by “use of false, misleading or deceptive forms,” “violent and aggressive behaviour towards the debtor” and “harassment.” These are corroborated by the findings of another recent study (Jérusalmy et al. 2020, p. 33–37). In the absence of clear rules regarding added charges, this remains a matter of utmost significance for all EU stakeholders involved in the debt collection process. As revealed by answers and comments of respondents, in many cases, these debt collection fees are passed from the creditor to the debtor, outside the contractual framework, which increases the level of consumer indebtedness artificially. While some debt-collector associations do not see this as an abusive practice, but an additional incentive or pressure for the consumer to pay her debts (France), others expressed concern about it and its effects on the consumers (Bulgaria, Czech Republic, and Germany).

Due to the absence of specifically designed remedies, the traditional ones—such as tort (civil), administrative or criminal liability—seem to play only a minor role in tackling abusive debt collection and cannot adequately protect consumers. In most cases, these procedures appear limited in scope, take too long, and are too expensive to be attractive to aggrieved consumer-debtors. As revealed by other empirical studies, costs associated with legal action taken by consumer will involve judicial fees, court expenses, and lawyer fees, which taken together constitute a major impediment to access to justice in consumer disputes (Caponi and Novak 2019, p. 69–70, 74). Moreover, respondent debt-collector associations reported that none of their members has incurred a criminal penalty, which seems to indicate that criminal actions taken via the state apparatus are not more attractive to consumers. Nevertheless, in the absence of clear numbers regarding civil or criminal cases against debt-collectors, the conclusion may be subject to revision.

## Conclusions

The results of this empirical study paint a more accurate and updated image of debt-collection regulation and its alternatives across the EU. As such, the study serves to expand and advance research and knowledge in the field and can provide the empirical foundation needed by EU policy makers to amend the NPLD Proposal (CEMA 2019).

Nevertheless, there are limits and potential shortcomings inherent to quantitative research. The most obvious is the complete absence of data for one MS: Malta, and the partial data for several MS. Although information was obtained from reliable respondents, such as competent state bodies (consumer or supervisory agencies), there is a risk that some answers were affected by the personal knowledge or understanding of their employees, as well as by their incomplete data. Concerning answers received from debt-collector associations, there is a risk of biased and inaccurate information.

These risks were mitigated by corroborating the answers among multiple respondents from the same MS, or with prior studies that covered similar topics. However, corroboration was not always possible. One should note that while quantitative research can help paint a more holistic picture of *how* abusive debt collection practices are regulated across the EU, it cannot explain *why* the situation presents itself as such. This remains a task for future qualitative research.

The study revealed a rather fractured and diverse legal framework for tackling abusive debt collection practices in the EU. Only one third of MS resort to sector-specific legislation, while the remaining ones have either scattered or no rules at all. Traditional remedies display idiosyncratic features and are affected by inherent shortcomings, all of which negatively impact their efficiency, harmonization of consumer financial protection, legal certainty, and predictability. If one can navigate through its national legal system, the situation changes dramatically in a cross-border setting, where additional barriers, such as language and legal traditions, are in place.^32^

This study confirms the initial hypothesis concerning the need for a sector-specific regulation of abusive debt collection practices. It also substantiates the opinion of stakeholders consulted about the NPLD Proposal. Most of them emphasized the need to regulate and supervise debt collectors. Some advocated harmonizing the rules regarding taxation and debtor protection (CEMA 2019). Some focused on punctual issues, such as debt collector’s remuneration schemes, qualification requirements, and respect for local rules, clear debt collection guidelines (Jérusalmy et al. 2020, pp. 4, 37).

The conclusion is that in the absence of convincing, converging, and widespread national solutions, the answer to the stakeholders’ needs is harmonization of regulation of abusive debt collection practices at EU level.

## Footnotes

1 The unemployment rate is 7.2 in EU 27. According to Eurostat, the unemployment rate caused by the Covid 19 is more severe than that caused by the financial crisis. Retrieved from https://ec.europa.eu/eurostat/statistics-explained/images/4/47/Unemployment_rates_EU-28%2C_EA-19%2C_seasonally_adjusted%2C_January_2000_-_July_2020.png (accessed 28 September 2020).

2 In *Commission of the European Communities v Italian Republic,* Case C-134/05, ECLI:EU:C:^2007^:435 *(Commission* vs. *Italy)*, Advocate General Maduro defined non-judicial debt collection as “any act or practice which is intended to encourage a debtor to settle an unpaid debt, with the exception of any recovery on the basis of an enforcement order.”

3 Retrieved from https://eur-lex.europa.eu/legal-content/EN/TXT/PDF/?uri=CELEX:52018PC0135&from=EN (accessed 30 July 2020).

4 Committee on Economic and Monetary Affairs’s Draft Report, of 29.11.2019, 2018/0063A (COD).

5 Art 114 corroborated with Art 169 of TFEU.

6 Art 3 (1) of UCPD 2005.

7 Directive (EU) 2019/2161 of the European Parliament and of the Council of 27 November 2019 amending Council Directive 93/13/EEC and Directives 98/6/EC, 2005/29/EC, and 2011/83/EU of the European Parliament and of the Council as regards the better enforcement and modernization of Union consumer protection rules [2019] OJ L238/7 (Enforcement Directive 2019).

8 For instance, in Reifner et al. (2010), it is stated that Denmark does not have a sector-specific law for debt collection, although it had implemented one in 1997. Tajti’s (2019) comparative assessment covers Hungary’s soft-law approach in wide detail and with anecdotal evidence; however, the rest of the information provided is lacunar and affected by the general absence of local sources in English or other large circulation language. For this reason, a number of key regulations and guidelines were omitted from the analysis. Finally, Reifner at al. (2010), Stănescu (2015) on Romanian law, Deville (2015) on UK law are currently outdated.

9 The starting point in the selection of respondents was the Consumer Protection Cooperation Network (CPCN)’s list of all authorities responsible for enforcing consumer protection laws in the EU and EEA countries. Only agencies that had competences concerning financial services, consumer credit, and unfair commercial practices were selected, resulting an initial number of eighty-five respondents. To these, I added the European Consumer Centres Network (ECCN)’s members, given their competence in settling cross-border consumer disputes, and those of the Bureau Européen des Unions de Consommateurs (The European Consumer Organization known as ‘BEUC’) that listed an email address through which they could be contacted. This raised the number of initial respondents by twenty-seven (one for each EU MS, respectively by forty (agencies from non-EU states affiliated to BEUC, were excluded from the scope of the survey). All these were contacted in the first phase of data collection (November 2019–January 2020). In a later stage of data collection, a number of courts with competences in supervising debt collection in the ‘lands’ of Germany were added to the list, which resulted in a total of one hundred and sixty seven institutions contacted.

10 Romania and Spain did not answer the survey either; however, they responded to a number of questions after petitions for access to information of public interest were filed by the author. These answers were considered in the final results of the survey.

11 The starting point for the list of respondents from the debt collection industry was the Federation of European National Collection Associations (FENCA)’s membership repository, consisting of national associations and affiliate members. Solely the associations and members from the surveyed MS and the directorate of FENCA have been contacted (sixty-nine respondents).

12 Retrieved from https://ec.europa.eu/info/sites/info/files/justice_scoreboard_2018_en.pdf (accessed 15 May 2020).

13 Retrieved from https://ec.europa.eu/newsroom/just/item-detail.cfm?item_id=59332 (accessed 15 May 2020).

14 Retrieved from https://europa.eu/european-union/about-eu/eu-languages_en (accessed 5 May 2020.

15 Although this stream of questions could have been dependent on the existence or not of a sector-specific legislation, a decision was made to allow respondents to see it because it was possible that such rules existed in other pieces of legislation. Nevertheless, as I point out in the Data Analysis, the responses obtained need to be taken cautiously.

16 The questions were posed to all respondents, notwithstanding whether their MS had a sector-specific legislation or not. On the one hand, the expectation was that those MS with such legislation in place will refer their answers mainly to the remedies and enforcement options provided by the said legislation. On the other hand, the survey aimed to gather data not only on the provisions of sector-specific legislation (estimated to be a minority at the time of the survey’s design), but also on general provisions and remedial options available to consumers in states with no sector-specific legislation as well.

17 According to data provided by Eurostat concerning the population in the 27 EU Member States at 1st January 2019. Retrieved from https://ec.europa.eu/eurostat/databrowser/view/tps00001/default/table?lang=en (accessed 10 May 2020).

18 In case of Netherlands, two respondents answered positively, while one answered negatively. The positive answer was chosen because it could be corroborated with the Netherlands Authority for Consumers and Markets’ Study into the Commercial Practices of Debt Collection Agencies, November 2015.

19 Not all answers received were consistent. For instance, among the answers received from Germany, several indicated ‘I do not know’ and only one indicated ‘Yes.’ Similarly, was the case for Netherlands or Ireland, where several answers indicated ‘No,’ respectively, ‘I do not know.’ However, for the purposes of this question, preference was given to clear answers; thus, a single answer of ‘Yes’ or ‘No’ was given preference over multiple ‘I do not know’ answers, to alleviate the risk of personal lack of knowledge.

20 Guidance on the Implementation of Directive 2005/29/EC on Unfair Commercial Practices, Brussels, 25.5.2016, COM (2016) 320 final (European Commission 2016), p. 7. Retrieved from https://eur-lex.europa.eu/legal-content/EN/TXT/PDF/?uri=CELEX:52016SC0163&from=EN (accessed 4 February 2020).

21 Most MS that regulate abusive debt collection practices combine licencing and supervision with bans of specific abusive practices (i.e., Belgium, Denmark, Finland, Romania, and Sweden). Other MS which do not have sector-specific legislation in place only require debt-collectors to hold a licence and thus be subjected to general supervision from state bodies (i.e., Austria, Italy). In both cases, failure to meet set-up and occupational standards may result in refusal of licence. For this reason, in the former’s case, it may be stated that licencing constitutes a *first line* of defence, while in the latter’s case it represents *the only* one.

22 It should be mentioned that German respondents provided contradictory answers, from which I considered the positive one was given by several institutions. The Italian respondent provided a negative answer, despite public information to the contrary, for which reason Italy is still counted here. Slovakia also provided contradictory answers, but since there was no possibility to further corroborate one of them, it was counted as unclear.

23 In the case of Austria, the respondent agency responded in the negative, while the debt-collection association responded positively and provided a copy of the national law concerning licencing. Thus, for the purposes of this question, the answer of the respondent agency was disregarded. In the case of Italy (where the answer to the survey was also negative), the licence requirement can be easily corroborated with other data. See *Commission of the European Communities v Italian Republic*, Case C-134/05, ECLI:EU:C:^2007^:435.

24 Most respondents from German state bodies have indicated a negative response. However, I have decided to count it among the affirmative answers, due to a high likelihood that civil remedies are present in general civil law, even if this position should be further corroborated with other sources to ensure absolute certainty.

25 Art 13 of Directive 2005/29/EC.

26 In 2018, the Swedish group Intrum has spent approximately 1.2 billion EUR in purchasing portfolios of overdue receivables with a return rate of 15%. Intrum’s 2018 Report, p. 13, retrieved from https://www.intrum.com/media/5068/intrum_asr_2018_en.pdf (accessed 7 January 2020). In 2019, the German group EOS announced a 668 million EUR investment in receivables and real estate and reported for 2018–2019 an increase in earnings (before interest, tax, depreciation, and amortization) of 283.6 million EUR, stemming mainly from purchases of unsecured and secured debt portfolios. EOS Group once again increases revenue and profit, 18.07.2019, retrieved from https://www.eos-solutions.com/Pool-Pressreleases.html?id=5ab0abeb-9d4b-4aad-b22a-1f22024cd763 (accessed 7 January 2020). Finally, the Polish group Kruk reported figures that are no less impressive. In the first three quarters of 2019, the company has spent approximately 92 million EUR on debt portfolios with a nominal value of approximately 946 million EUR and recovered almost 308 million EUR. Report no 47/2019: Expenditure on and recoveries from debt portfolios. Retrieved from https://en.kruk.eu/investor-relations/reports/current-reports/current-report-no-47-2019-expenditure-on-and-recoveries-from-debt-portfolios (accessed 7 January 2020).

27 A complete list of ADR bodies available in each Member State can be found at: https://www.evz.de/en/shopping-internet/alternative-dispute-resolution/adr-bodies-in-the-eu.html (accessed 13 May 2020).

28 Commission Staff Working Document Impact Assessment—The development of secondary markets for non-performing loans by removing undue impediments to loan servicing by third parties and the transfer of loans (Part 1/2) and accelerated extrajudicial collateral enforcement (Part 2/2), Brussels, 14.03.2018, SWD (2018) 75 final, Part ½ (Impact Assessment), p. 77. Retrieved from http://europeanmemoranda.cabinetoffice.gov.uk/files/2018/03/ST-7403-2018-ADD-1-EN.pdf (accessed 30 June 2020).

29 It should be stated here that the NPLD Proposal speaks of loan servicers, a category wider than that of debt collectors. Thus, I do not exclude that some other categories of loan servicers, such as debt buyers, face difficulties in providing their services outside their home jurisdiction. However, the same cannot be said about debt collectors.

30 *Supra,* fn 2.

31 Ibid., 76–77.

32 Legal traditions and heterogeneous consumer protection rules are mentioned among obstacles in the NPLD Proposal’s Impact Assessment (p. 74) but also in the proposed amendments via CEMA’s Draft Report (2019). As another pan-EU empirical study revealed, “court processes are […] certainly not attractive in the cross-border situation” (Hodges 2019, p. 198).
